# Cellular and Systemic Toxicity Induced by Sunscreen and Paraben Mixtures and the Implications for Root Development in Cultivated Plants

**DOI:** 10.1155/jt/7171333

**Published:** 2026-04-28

**Authors:** Ana Elisa Maehashi, Emily de Moura Galdino, Edson Araujo de Almeida, Diego Espirito Santo, Carmem Lúcia Henrich, Bianca da Cruz, Elisângela Dusman, Danielle Cristina da Silva de Oliveira, Gideã Taques Tractz, Regiane da Silva Gonzalez, Osvaldo Valarini Junior, C. A. Downs, Ana Paula Peron

**Affiliations:** ^1^ Chemical Engineering Course, Federal Technological University of Paraná, Campo Mourão, Paraná, Brazil, utfpr.edu.br; ^2^ Postgraduate Program in Technological Innovations, Federal Technological University of Paraná, Campo Mourão, Paraná, Brazil, utfpr.edu.br; ^3^ Postgraduate Program in Chemistry, Maringá State University, Maringá, Paraná, Brazil, uem.br; ^4^ Postgraduate Program in Biological Sciences, State University of Londrina, Londrina, Paraná, Brazil, uel.br; ^5^ Postgraduate Program in Environmental Engineering, Federal Technological University of Paraná, Francisco Beltrão, Paraná, Brazil, utfpr.edu.br; ^6^ Academic Department of Chemistry, Federal Technological University of Paraná, Campo Mourão, Paraná, Brazil, utfpr.edu.br; ^7^ Postgraduate Program in National Network in Management and Regulation of Water Resources, Federal Technological University of Paraná, Campo Mourão, Paraná, Brazil, utfpr.edu.br; ^8^ Postgraduate Program in Food Technology, Federal Technological University of Paraná, Campo Mourão, Paraná, Brazil, utfpr.edu.br; ^9^ Haereticus Environmental Laboratory, Gladstone, Virginia, USA

**Keywords:** cultivated plants, cytogenotoxicity, micropollutants in mixture, oxidative stress, phytotoxicity, productivity

## Abstract

Sunscreens and parabens contaminate agricultural areas worldwide; however, the effects of these micropollutants in mixture on cultivated plants have not yet been reported. This study evaluated the cellular and systemic toxicity induced by octocrylene (OC), methylparaben (MeP), and butylparaben (BuP), individually at concentrations of 10, 50, 100, and 500 ng·L^−1^, as well as by equimolar binary mixtures (1:1) of OC with MeP and OC with BuP, in seeds of *Cucumis sativus* L. and *Lycopersicum esculentum* L., and in roots of *Allium cepa* L. bulbs. The OC + MeP mixtures induced H_2_O_2_ accumulation, whereas the OC + BuP mixtures promoted lipid peroxidation in the root meristems of *A. cepa*, resulting in significant mitodepressive, aneugenic, and clastogenic effects. The OC + BuP combination markedly reduced root growth in the three evaluated species and, in onion, caused mitotic indices below 50% compared to the control, demonstrating severe cytotoxicity to the meristems. In contrast, the OC + MeP combination stimulated root growth in cucumber, tomato, and onion. However, although significantly longer, the formed roots exhibited greater susceptibility to breakage than the control, indicating that growth predominantly associated with cell elongation, without concomitant cell proliferation. The interaction between the compounds was characterized as synergistic for OC + MeP and additive for OC + BuP, with BuP being the main determinant of mixture toxicity. Thus, mixtures of OC + MeP and OC + BuP may impair the early establishment of crops by affecting root system functionality, delaying or inhibiting root development, and altering root structural stability. These effects indicate that the presence of these micropollutant mixtures in contaminated agricultural soils may negatively influence plant development and the agronomic performance of cultivated plants.

## 1. Introduction

Alkyl esters derived from 4‐hydroxybenzoic acid, commonly referred to as parabens, have been employed for over 5 decades as antimicrobial preservatives in food, pharmaceutical, and cosmetic formulations, with an estimated annual industrial production exceeding 8000 tons [1–4]. Among them, methylparaben (MeP) and butylparaben (BuP) are the most widely used worldwide because of their broad‐spectrum activity against fungi and bacteria, adequate solubility, chemical stability under different temperatures and pH conditions [5–7], and relatively low acute toxicity in mammals [2, 3, 8]. In addition, these compounds are characterized by a lack of noticeable odor or color and low volatility [9].

Octocrylene (OC) is a sunscreen ingredient used in cosmetics and pharmaceuticals, with the ability to absorb ultraviolet B radiation in the 280–320‐nm range and enhance the stability of avobenzone [10, 11]. In 2024, the global OC market was valued at US$266.6 million, the largest among sunscreens, and is estimated to reach US$403.4 million by 2033 [12]. This filter is a colorless, lipophilic, high‐molecular‐weight cinnamate ester [13, 14].

Lotions, creams, ointments, and gels marketed internationally contain parabens and sunscreens, with OC, MeP, and BuP being the most frequently detected [15, 16]. These compounds are classified as micropollutants due to the lack of regulations governing their disposal in the environment, mainly because of the limited number of studies on their impacts across different ecosystems [10, 17–20]. OC is known to be recalcitrant in water and soil and highly resistant to photodegradation [13, 17, 21, 22], whereas MeP and BuP exhibit relatively rapid biodegradation in these compartments, with an average half‐life of approximately 15 days. However, they are classified as pseudo‐persistent because they are continuously discarded into the environment [23–25].

In agricultural soils, the occurrence of OC, MeP, and BuP is mainly associated with the application of sewage sludge as fertilizer and the reuse of treated wastewater for crop irrigation. These practices have enabled the detection of such compounds at concentrations ranging from ng·L^−1^ to µg·L^−1^ in soil and irrigation matrices [5, 7, 26–28]. Experimental studies report that OC and MeP at µg·L^−1^ levels and BuP at ng·L^−1^ concentrations induce significant cellular and systemic toxicity in plants and earthworms [8, 9, 11, 19, 20], as well as toxic and endocrine‐disrupting effects in soil nematodes [29].

The average annual global production of domestic sewage sludge is approximately 45 million megagrams of dry matter. More than half of this amount is used in agriculture as fertilizer [30, 31]. Moreover, in economically developing countries, as well as in arid regions, urban wastewater constitutes a substantial proportion of the water used for irrigation of adjacent agricultural areas [31–33]. Although contamination of agricultural soils by micropollutants is widespread and recurrent, studies on sunscreens and parabens and their impacts on agricultural productivity remain incipient. It should be noted that the evidence available in the literature on the impacts of micropollutants on the development of crops refers almost exclusively to microplastics [34, 35].

Studies evaluating the adverse effects of micropollutants in isolation are relevant and form the basis of available ecotoxicological knowledge. However, these approaches prove insufficient for understanding the real environmental risks, since these xenobiotics coexist in water and soil and can interact additively, synergistically, or antagonistically [15, 36, 37]. In turn, environmental toxicological analyses of emerging contaminants in mixtures currently available focus mainly on aquatic species, while the potential impacts of these exposures on cultivated plants are scarce [38–40]. To the best of our knowledge, no ecotoxicological studies have evaluated mixtures of organic sunscreens and parabens, regardless of the trophic level considered.

The use of domestic sewage sludge and other animal‐derived wastes as fertilizers in agriculture is a concern in countries that adopt this practice, as these materials may contain pathogenic microorganisms. To mitigate bacterial contamination, the Food and Drug Administration [41] and the National Agricultural Law Center [42] consider, albeit at a conceptual level, the use of parabens in sludge and agricultural soils as part of an integrated pathogen management plan for leafy vegetables. In this context, the continuous contamination of agricultural soils and the potential use of parabens as antimicrobials in cultivated soils and/or sludge reinforce the urgency of evaluating the ecotoxicological impacts of these micropollutants, in mixtures with other contaminants, on crops and key species within agricultural landscapes.

*Cucumis sativus* L. (cucumber) and *Lycopersicum esculentum* L. (tomato) are plant biomodels widely recognized in ecotoxicological research. They are recommended by the United States Environmental Protection Agency (USEPA) [43] and the Organization for Economic Co‐operation and Development (OECD) [44] for assessing the adverse effects of environmental contaminants. These species allow the evaluation of sublethal endpoints, including effects on germination and meristematic tissues [8, 19]. The root meristems of *Allium cepa* L. (onion) have also been used for more than 5 decades as a sensitive bioassay system, enabling the detection of morphological, physiological, cytogenetic, and biochemical alterations induced by chemical agents [4, 28, 45, 46]. Results obtained with this test are consistent with findings in other biological systems, including animal models and cell cultures, supporting its reliability for assessing phytotoxicity, cytotoxicity, and genotoxicity [4, 11, 28, 33, 45–48].

Therefore, to contribute to the understanding of the ecotoxicological risks associated with the presence of sunscreens and parabens in agricultural environments, the objective was to evaluate the cellular and systemic toxicity induced by OC in binary mixtures with MeP and BuP in *C. sativus*, *L. esculentum*, and *A. cepa*. Toxic effects were investigated through an integrated assessment encompassing growth responses, cellular alterations, and biochemical biomarkers of oxidative stress. For comparative purposes and to assess toxic interactions among these micropollutants, the effects of OC, MeP, and BuP in isolation were evaluated under the same experimental conditions as the mixtures, using morphological, physiological, and cytogenetic parameters.

The present study expands the ecotoxicological investigation previously conducted by our group on ethylparaben applied individually [49] by addressing mixture toxicity involving different micropollutants under environmentally relevant conditions.

## 2. Material and Methods

### 2.1. Obtaining OC, MeP, and BuP, Defining and Preparing Concentrations for Study

The micropollutants OC (2‐ethylhexyl‐2‐cyano‐3,3‐diphenylacrylate, CAS 6197‐30‐4, molecular weight 361.48 g·mol^−1^, and log Kow 6.88), MeP (methyl 4‐hydroxybenzoate, CAS 99‐76‐3, molecular weight 152.15 g·mol^−1^, and log Kow 1.96), and BuP (butyl 4‐hydroxybenzoate, CAS 94‐26‐8, molecular weight 194.23 g·mol^−1^, and log Kow 3.57) were obtained in analytical grade from Sigma‐Aldrich, as were the other reagents used in this study.

In different countries, OC has been detected in urban sludge and reused water in concentrations ranging from 212.9 ng·L^−1^ to 500 µg·L^−1^ [11, 18, 32]. In comparison, MeP and BuP were found in soil at concentrations ranging from 101.6 ng·L^−1^ to 501.4 µg·L^−1^, in domestic sewage sludge from 27 ng·L^−1^ to 107 µg·L^−1^, and in treated wastewater from 10.1 to 50.9 µg·L^−1^ [20, 50–53].

Although concentrations of OC, MeP, and BuP reported in the literature range from ng to µg levels, these compounds were evaluated at ng·L^−1^ levels to reflect their levels in agricultural soils better. The three micropollutants were assessed individually (10, 50, 100, and 500 ng·L^−1^) and as equimolar binary mixtures (1:1), corresponding to the combinations of OC + MeP and OC + BuP at the same nominal concentrations (OC 10 + MeP 10, OC 50 + MeP 50, OC 100 + MeP 100, OC 500 + MeP 500, OC 10 + BuP 10, OC 50 + BuP 50, OC 100 + BuP 100, and OC 500 + BuP 500).

OC, MeP, and BuP are low‐polarity compounds and therefore have low solubility in water. To ensure the solubility of these compounds in aqueous solution, isolated concentrations and mixtures were prepared in an aqueous medium using the surfactant Tween 80 at a mass concentration equivalent to that of the micropollutants. Tween 80 is a surfactant of very low toxicity and is widely used in ecotoxicological studies involving organic pollutants [3, 4, 8].

To ensure that any observed biological effects were not attributable to the surfactant, a specific Tween 80 control was included in all biological assays. The concentration used for this control was 500 ng·L^−1^, corresponding to the highest concentration employed for the micropollutants.

The term “treatment(s)” refers to the control, isolated concentrations, and mixed concentrations.

### 2.2. Stability Analysis of OC, MeP, and BuP in Aqueous Media

OC, MeP, and BuP stock solutions, at a concentration of 0.03 g·L^−1^, were prepared to evaluate the individual stability of these compounds in an aqueous medium, in the absence of light and over 7 days, a period corresponding to the exposure time of the seeds and roots to the treatments (as described in items 2.3.1 and 2.3.2). The analyses were performed on a UV–vis spectrophotometer at 305 nm for OC, 255 nm for MeP, and 255 nm for BuP. The results were calculated according to (1) and expressed as a percentage relative to time zero.

(1) Stability%=sample absorbance absorbance day zero ×100.

### 2.3. Plant Assays for the Evaluation of Micropollutants Individually and in Mixtures

#### 2.3.1. Evaluation of Phytotoxicity in Seeds of *C. sativus* and *L. esculentum*

For this evaluation, commercially purchased, nontransgenic, agrochemical‐free seeds of *C. sativus* and *L. esculentum* were used. According to the manufacturer’s information, the seeds had a germination rate greater than 95% and a purity of 96%–99%.

The phytotoxic potential was determined using germination and radicle elongation parameters, according to the OECD protocol [44]. Seeds from each vegetable were distributed equidistantly in Petri dishes that had been previously sterilized and lined with filter paper. Twenty seeds were used per dish, and each treatment was evaluated in quintuplicate. In each dish, the filter paper was soaked with 1.5 mL of its respective treatment. The dishes were then sealed with plastic film and placed in a biochemical oxygen demand (BOD) incubator at 25°C in the dark for 7 days. Every 48 h, the need to replace the treatment solutions was assessed and, when necessary, 0.5 mL of the corresponding solution was added. Distilled water was used as a control.

After 7 days, the germination percentage (*G*%) of each treatment was calculated (equation (2)). A seed was considered germinated after the emergence of the radicle.

(2) G%:number of seeds germinated number of seeds evaluated ×100.

After 7 days, the rootlets were measured with a caliper, and the Relative Growth Index for each treatment was calculated.

(3) RGI=RLIRLC,

where RGI corresponds to the Relative Growth Index, RLI to the average length of roots exposed to treatment, and RLC to the average length of control roots.

According to Biruk et al. [54], RGI values between 0.8 and 1.2 indicate that root growth was not affected, while values below 0.8 characterize inhibition of root elongation. Values above 1.2 indicate stimulation of root growth.

In addition, the morphology, coloration, and susceptibility to breakage of the roots were evaluated.

#### 2.3.2. Analysis of Phytotoxicity, Cytotoxicity, and Genotoxicity in Roots of *A. cepa* Bulbs

Cellular and systemic toxicity analyses on *A. cepa* bulb roots were conducted according to Fiskesjö [55], with adaptations by Nascimento et al. [19] and Filipi et al. [56]. The onion bulbs were purchased from an organic produce store. Initially, the dry cataphylls were removed, and the bulbs were washed in distilled water. After cleaning, the onions were placed in beakers containing the treatment solutions, with the basal region of the bulbs submerged. The treatment solutions were prepared and renewed daily, always at the same time. The beakers containing the onions were kept in a BOD chamber at 25°C in the dark for 7 days. Each treatment was analyzed in quintuplicate. Distilled water was used as the negative control. Methyl methanesulfonate (10 µg·L^−1^) was used as the positive control, according to Dias et al. [45].

After 7 days, the length of 10 roots from each bulb was measured with a caliper, and the average root length (ARL) was calculated for each treatment (equation (4)).

In addition, the morphology, coloration, and susceptibility to breakage of the roots were evaluated.

(4) ARLcm: Sum of root length of root bundles10.

For cytotoxicity and genotoxicity analysis, roots from each onion (an average of 5 roots per bulb) were collected and fixed in Carnoy’s solution (3:1, v/v) for at least 12 h. After fixation, the meristematic regions of the roots were highlighted, and slides were prepared for analysis under an optical microscope at 400x magnification.

To determine cytotoxic potential, 10.000 cells per treatment (2.000 cells per bulb) were evaluated, and the Mitotic Index (MI) was calculated (equation (5)). To evaluate genotoxic potential, 2.000 cells per treatment (200 per bulb) were analyzed, and the Cellular Alteration Index (CAI) was calculated (equation (6)). Cell alterations included chromosomal disorganization and loss in the different phases of mitosis, anaphase and telophase bridges, sticky chromosomes, polyploidy, and micronuclei.

(5) MI:Total number of dividing cells10000,×100,

(6) CAI: Number of cellular alterations2000,×100.

### 2.4. Biochemical Analyses of *A. cepa* Bulb Roots Exposed to OC in a Mixture With MeP and BuP

#### 2.4.1. Preparation of Roots for Enzymatic Analysis

To obtain the enzyme extracts, 50 mg of root meristems (as described in Section 2.3.2) were collected from each bulb replicate. The meristems were macerated in 3 mL of potassium phosphate buffer (50 mM, pH 7.0) containing 5 mM DPTA, and the resulting homogenates were centrifuged at 4.000 rpm for 15 min at 4°C. This approach ensured that the enzymes were extracted under mild conditions that preserve their activity.

Enzyme extracts were analyzed for the activities of antioxidant enzymes catalase (CAT), ascorbate peroxidase (APX), guaiacol peroxidase (GPOX), and superoxide dismutase (SOD).

#### 2.4.2. Enzyme Analyses

The CAT activity was evaluated using the protocol described by Kraus et al. [57], with modifications by Azevedo et al. [58]. To the enzyme extract (100 µL) from each bulb repetition, 2.5 mL of sodium phosphate buffer (pH 7.8) and 1 mL of 1 mM H_2_O_2_ were added. The extracts were then read in a UV–vis spectrophotometer at 240 nm. The extinction coefficient was 2.8 M^−1^·cm^−1^, and the results were presented in µmol·min^−1^·µg^−1^ of protein.

(7) U=A/t/E×Ve×DFP,

where *U* is the enzyme unit, A is the measured absorbance, *t* is the analysis time, *E* is the extinction coefficient, *V*_*e*_ is the enzyme volume, DF is the dilution factor, and *p* is the protein, obtained from the mass of roots used.

APX enzyme activity was determined as described by Zhu et al. [59]. To the enzyme extract (100 µL) from each bulb repetition, 2.5 mL of sodium phosphate buffer, 500 µL of 0.25 mM ascorbic acid, and 1 mL of 1 mM H_2_O_2_ were added. The absorbance of the extracts was then read at 290 nm. The extinction coefficient was 2.8 M^−1^·cm^−1^, and the results were expressed in µmol·min^−1^·µg^−1^ of protein (7).

GPOX modulation was measured as described by Matsuno and Uritani [60]. In 300 µL of the enzyme extract from each bulb repetition, 2.5 mL of sodium phosphate buffer, 250 µL of 0.1 M citric acid, 250 µL of 0.5% guaiacol, and 250 µL of 1 mM H_2_O_2_ were added. The extracts were then homogenized and incubated at 30°C for 15 min, followed by placement in an ice bath for 10 min and the addition of 250 µL of 2% sodium metabisulfite. The absorbance of the extracts was measured in a UV–vis spectrophotometer at 450 nm. For these analyses, an extinction coefficient of 26.6 M^−1^·cm^−1^ was used, and the results were presented in µmol·min^−1^·µg^−1^ of protein (equation (7)).

SOD modulation was obtained according to the protocol of Sun et al. [61]. The enzyme extract from each bulb repetition was prepared in duplicate: half of the samples were exposed to an 80‐W fluorescent light for 20 min, and the other half were kept in the dark. A 200‐µL aliquot of each sample received 0.8 mL of sodium phosphate buffer, 500 µL of 0.1 mM EDTA, 500 µL of methionine, 500 µL of NBT, and 200 µL of riboflavin. The absorbance of the samples was measured at 560 nm in a UV–vis spectrophotometer, and enzymatic activity was expressed as units per protein.

(8) SOD=Bl−sl/Bl−Be−se/Be50,

where *B*_*l*_ is the absorbance of the blank kept in the light prepared without the enzyme extract, *s*_*l*_ is the absorbance of the sample kept in the light, *B*_*e*_ is the absorbance of the blank kept in the dark, and *s*_*e*_ is the absorbance of the sample kept in the dark. The quotient 50 represents the amount of enzyme required to inhibit 50% of the photoreduction of NBT.

#### 2.4.3. Analysis of Antioxidant Activity (DPPH), Phenolic Content (Folin–Ciocalteu (FC)), and Lipid Peroxidation (TBARS) in Root Meristems

##### 2.4.3.1. Sample Preparation

For each bulb repetition, 50 mg of root meristems (obtained in item 2.3.2) were removed, then mixed with 3 mL of distilled water, and centrifuged for 15 min at 4.000 rpm to obtain homogenates.

##### 2.4.3.2. DPPH Assay

The antioxidant potential was determined according to the protocol of Unalan et al. [62], in which 250 µL of a 0.00316% (m/V) DPPH solution was added to 50 µL of the homogenate from each bulb repetition. The homogenates were left to stand in the dark for 30 min and then evaluated in a UV–vis spectrophotometer at 515 nm. Antioxidant activity was calculated according to the following equation:

(9) A%=Ac−AsAc×100,

where AA% is the antioxidant activity, *A*_*c*_ is the absorbance of the DPPH solution without the sample, and *A*_*s*_ is the absorbance of the sample with DPPH.

##### 2.4.3.3. FC Assay

The phenolic content was evaluated according to the Carmona‐Hernandez [63] protocol. To 50 µL of homogenate from each bulb repetition, 50 µL of distilled water, 50 µL of ethanol, and 100 µL of FC (0.0288 g of phosphotungstic acid and 0.0182 g of phosphomolybdic acid dissolved in 5 mL of methanol) were added. The homogenates were then left at room temperature and in total darkness for 10 min. Subsequently, 50 µL of saturated sodium bicarbonate solution was added to the homogenates, which were left in the dark for 50 min. At the end of this period, the mixtures were read in a UV–vis spectrophotometer at 745 nm.

##### 2.4.3.4. TBAR Assay

Lipid peroxidation was evaluated according to the method of Papastergiadis et al. [64]. To the homogenates (50 µL) of each bulb repetition, 250 µL of TBAR solution (46 mM) was added, and the mixture was placed in a water bath at 90°C for 35 min. Immediately after cooling, the samples were evaluated in a UV–vis spectrophotometer at 532 nm.

### 2.5. Justification for Repeating Ecotoxicological Assessment Tests on BuP Alone

Although the micropollutant BuP alone has been investigated in a previous study by Tsubouchi et al. [65] for cellular and systemic toxicity in plants at concentrations of 10, 50, 100, and 500 ng·L^−1^, in the present study, these assessments were repeated to establish a comparative basis for interpreting the toxic effects resulting from the combined exposure to OC and BuP on the initial development of cultivated plants.

### 2.6. Statistical Analysis

The phytotoxicity, cytotoxicity, genotoxicity, and biochemical analysis data were analyzed using the nonparametric Kruskal–Wallis test, followed by Dunn’s test, with a significance level of *p* ≤ 0.05. These tests were chosen based on the non‐normality of the data, verified by the Lilliefors test. Statistical analyses were performed using RStudio software [66].

## 3. Results

### 3.1. Stability of OC, MeP, and BuP in Aqueous Media

To ensure the reliability of the exposure conditions, the chemical stability of OC, MeP, and BuP was assessed separately in aqueous solution over 7 days (Figure 1). The concentrations measured during the experimental timeframe showed only slight variations relative to the initial values, indicating that the micropollutants remained stable under the tested conditions.

FIGURE 1Stability of methylparaben (a), butylparaben (b), and octocrylene (c) in an aqueous medium for 7 days.

**Figure:**
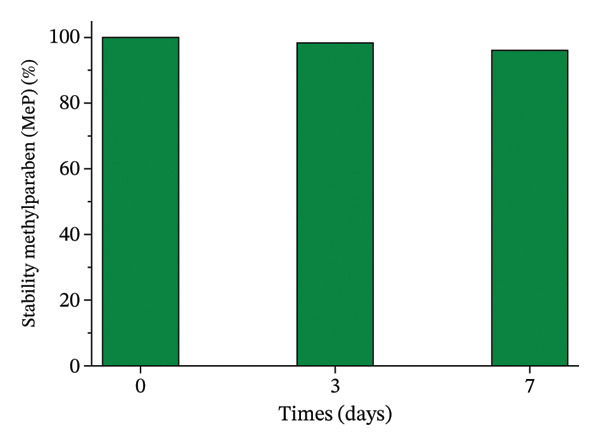
(a)

**Figure:**
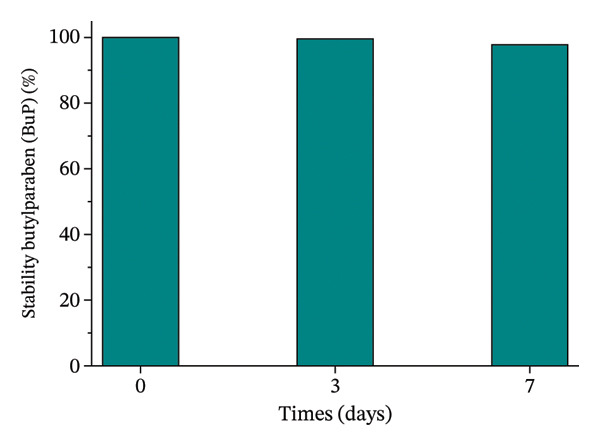
(b)

**Figure:**
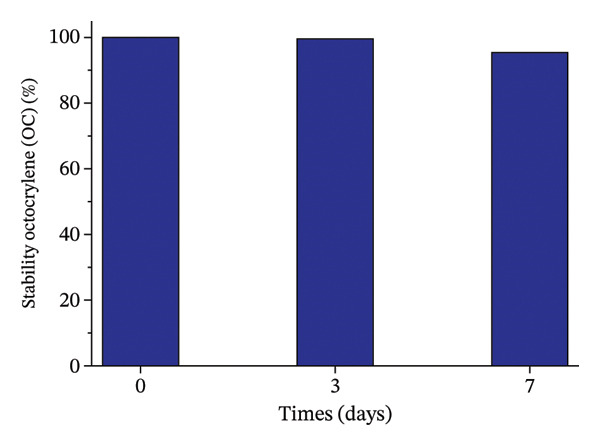
(c)

### 3.2. Phytotoxicity in Seeds of C. sativus and L. esculentum and in Roots of A. cepa

As shown in Figure 2, OC, MeP, and BuP, when evaluated individually, did not impair seed germination in *C. sativus* (Figures 2(b), 2(f), and 2(j)) and *L. esculentum* (Figures 2(d), 2(h), and 2(l)). In these species, isolated OC and MeP stimulated root growth, with RGI values greater than 1.2 (Figures 2(a), 2(c), 2(e), and 2(g)). At the same time, BuP caused a reduction in rootlet length (RGI < 0.8) (Figures 2(i) and 2(k)) at all concentrations evaluated. Cucumber and tomato roots treated with these compounds did not show alterations in color or morphology and maintained structural integrity upon handling.

FIGURE 2Phytotoxicity of octocrylene, methylparaben, and butylparaben, and of octocrylene in binary mixtures with methylparaben or butylparaben, on seeds of *Cucumis sativus* L. and *Lycopersicum esculentum* L. Seeds were exposed to concentrations of 10, 50, 100, and 500 ng·L^−1^. Effects were evaluated using the parameter seed germination and relative growth index. ^∗^Significant difference in relation to the control and ^#^significant difference between the binary mixtures and the respective isolated micropollutants, according to the Kruskal–Wallis H test, followed by Dunn’s post hoc test (*p* ≤ 0.05). OC‐octocrylene, MeP‐methylparaben, BuP–butylparaben, T80–Tween 80 (500 ng·L^−1^). (a) RGI obtained for OC (10, 50, 100, and 500 ng·L^−1^) in *C. sativus*; (b) G (%) obtained for OC (10, 50, 100, and 500 ng·L^−1^) in *C. sativus*; (c) RGI obtained for OC (10, 50, 100, and 500 ng·L^−1^) in *L. esculentum*; (d) G (%) obtained for OC (10, 50, 100, and 500 ng·L^−1^) in *L. esculentum*; (e) RGI obtained for MeP (10, 50, 100, and 500 ng·L^−1^) in *C. sativus*; (f) G (%) obtained for MeP (10, 50, 100, and 500 ng·L^−1^) in *C. sativus*; (g) RGI obtained for MeP (10, 50, 100, and 500 ng·L^−1^) in *L. esculentum*; (h) G (%) obtained for MeP (10, 50, 100, and 500 ng·L^−1^) in *L. esculentum*; (i) RGI obtained for BuP (10, 50, 100, and 500 ng·L^−1^) in *C. sativus*; (j) G (%) obtained for BuP (10, 50, 100, and 500 ng·L^−1^) in *C. sativus*; (k) RGI obtained for BuP (10, 50, 100, and 500 ng·L^−1^) in *L. esculentum*, (l) G (%) obtained for BuP (10, 50, 100, and 500 ng·L^−1^) in *L. esculentum*; (m) RGI obtained for OC + MeP (10, 50, 100, and 500 ng·L^−1^) in *C. sativus*; (n) G (%) obtained for OC + MeP (10, 50, 100, and 500 ng·L^−1^) in *C. sativus*; (o) RGI obtained for OC + MeP (10, 50, 100, and 500 ng·L^−1^) in *L. esculentum*; (p) G (%) obtained for OC + MeP (10, 50, 100, and 500 ng·L^−1^) in *L. esculentum*; (q) RGI obtained for OC + BuP (10, 50, 100, and 500 ng·L^−1^) in *C. sativus*; (r) G (%) obtained for OC + BuP (10, 50, 100, and 500 ng·L^−1^) in *C. sativus*; (s) RGI obtained for OC + BuP (10, 50, 100, and 500 ng·L^−1^) in *L. esculentum*; and (t) G (%) obtained for OC + BuP (10, 50, 100, and 500 ng·L^−1^) in *L. esculentum*.

**Figure:**
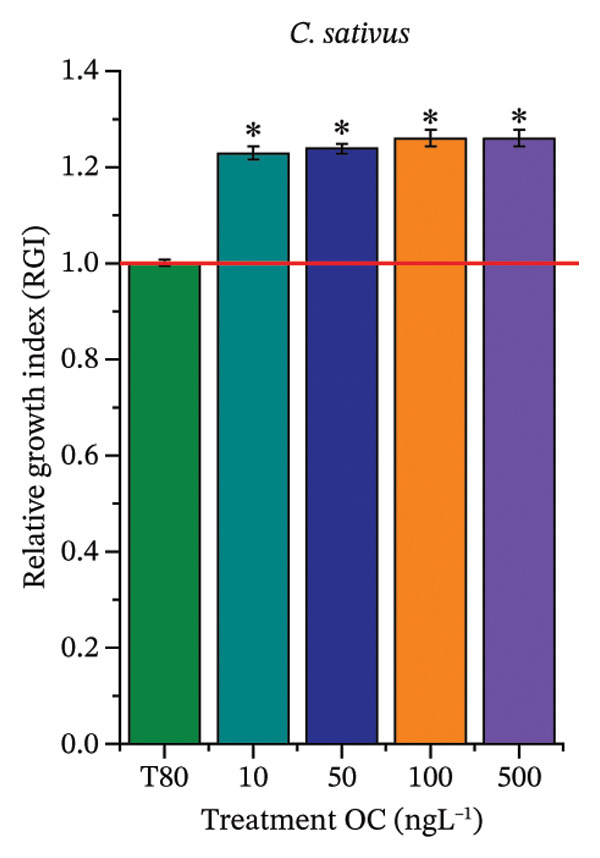
(a)

**Figure:**
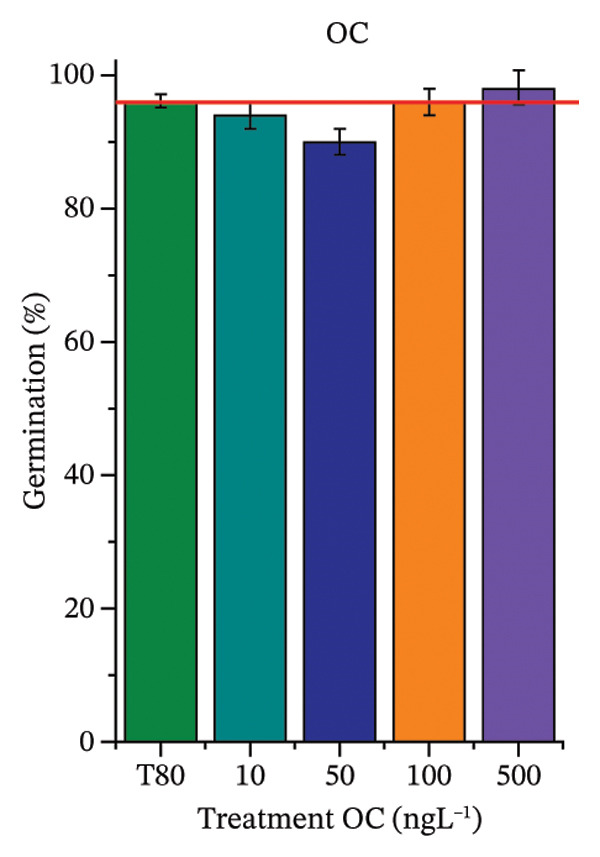
(b)

**Figure:**
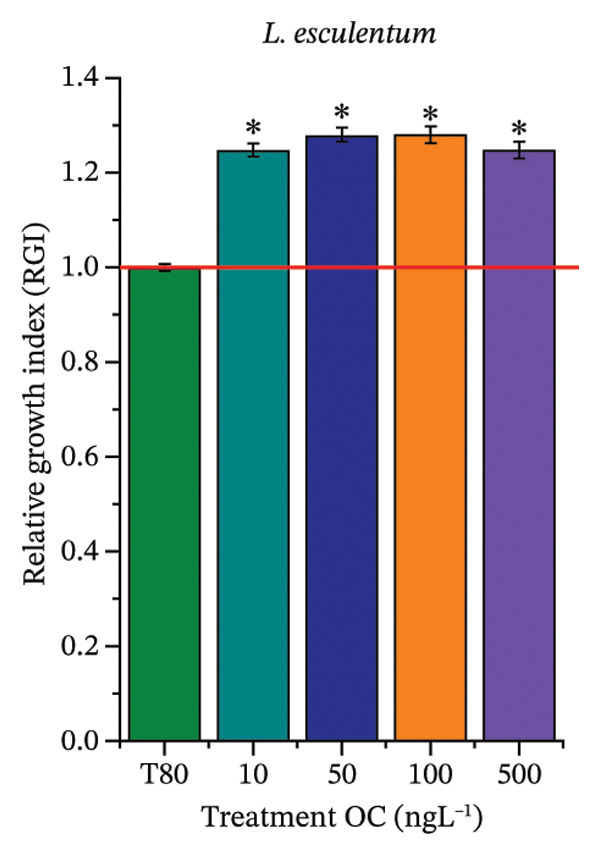
(c)

**Figure:**
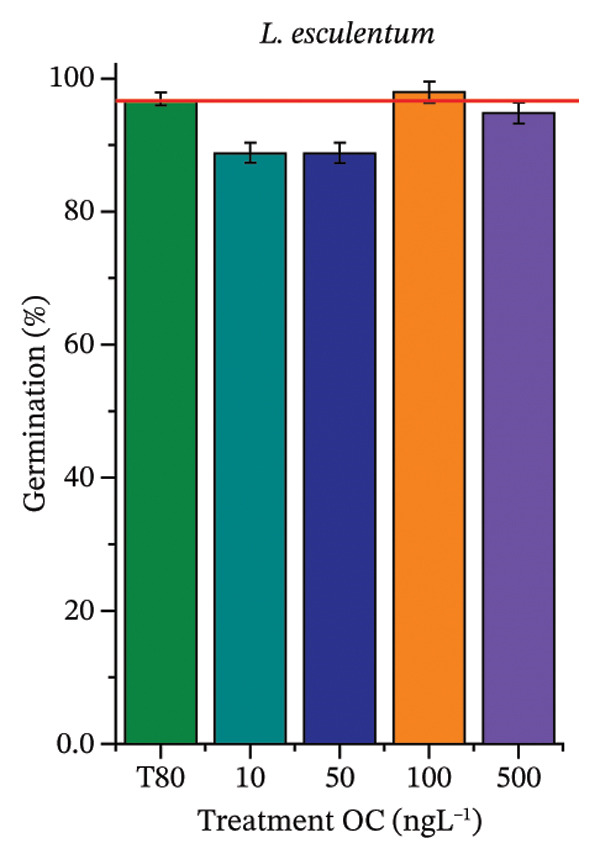
(d)

**Figure:**
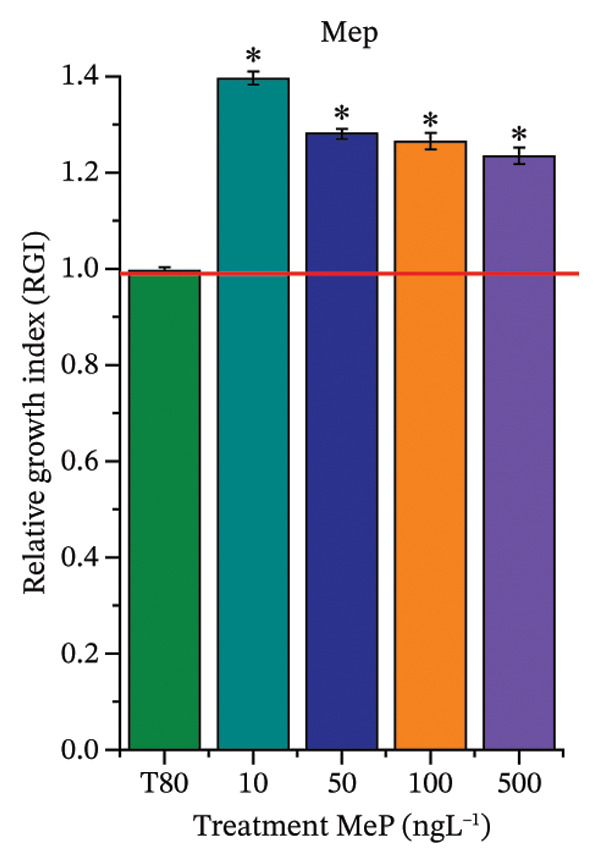
(e)

**Figure:**
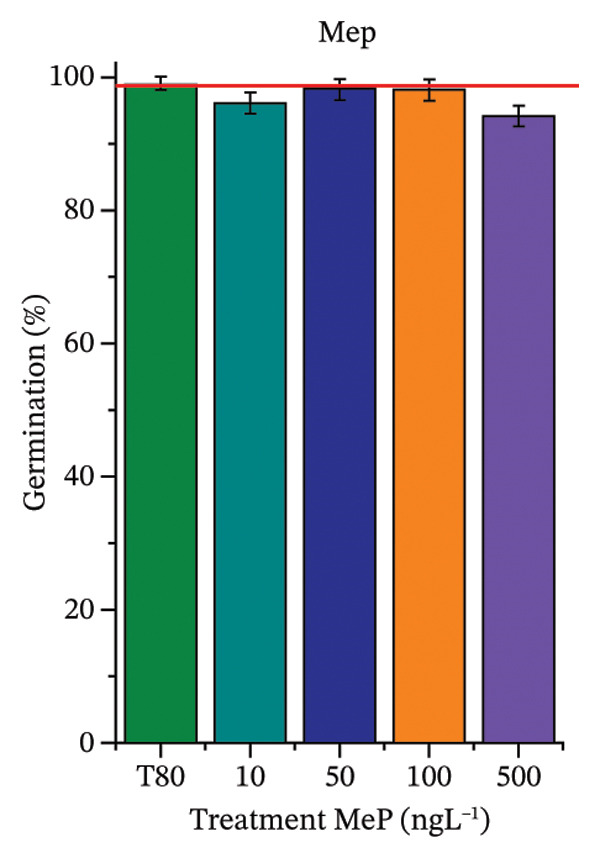
(f)

**Figure:**
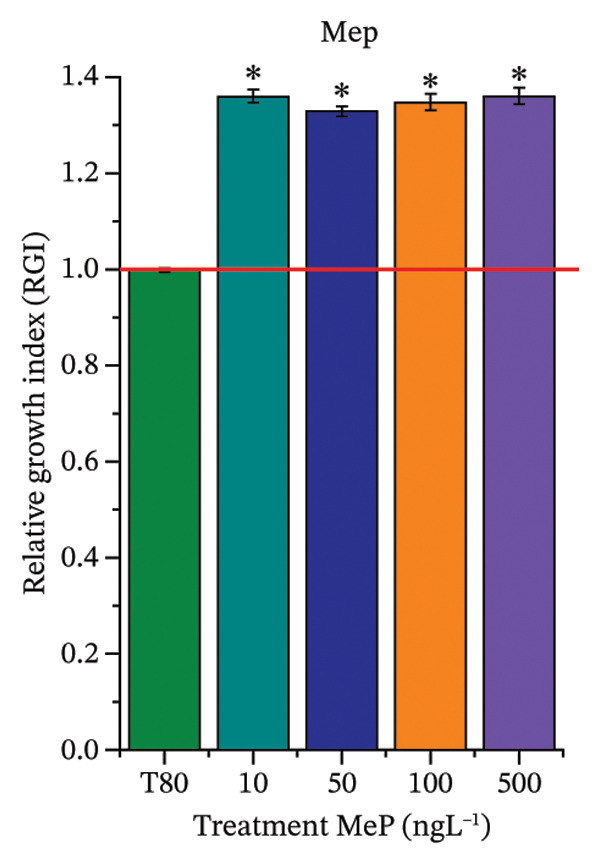
(g)

**Figure:**
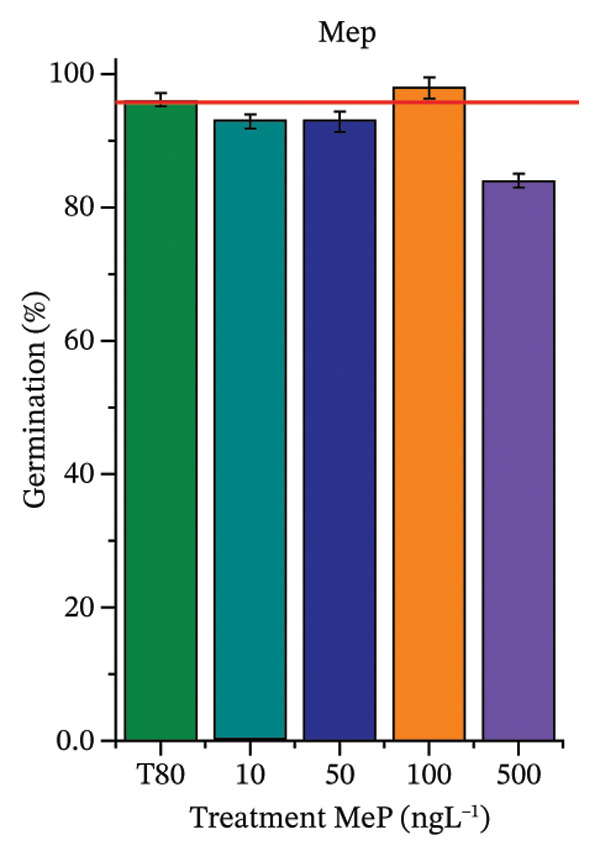
(h)

**Figure:**
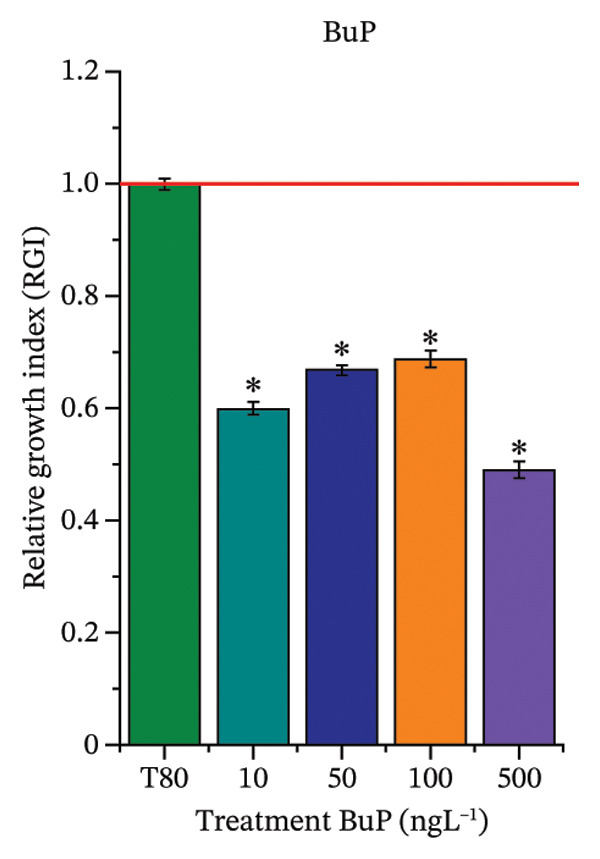
(i)

**Figure:**
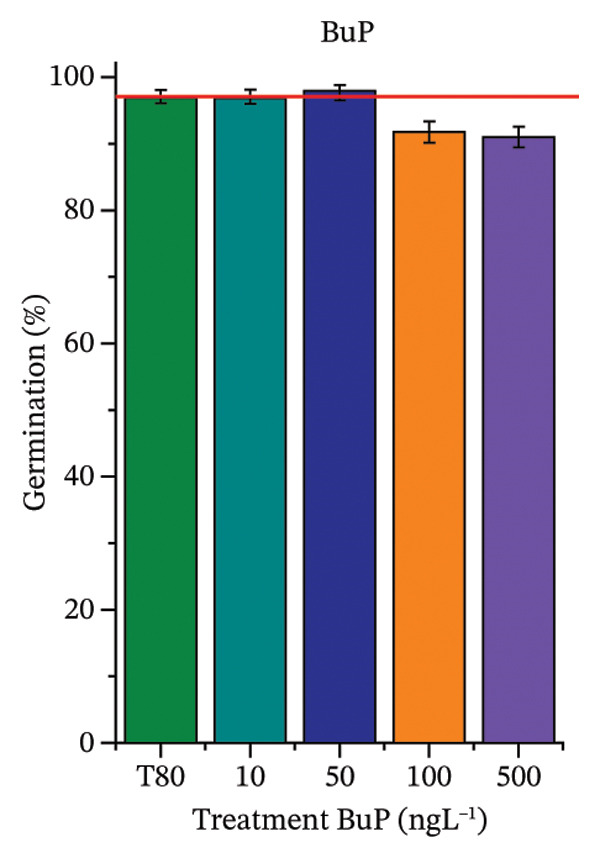
(j)

**Figure:**
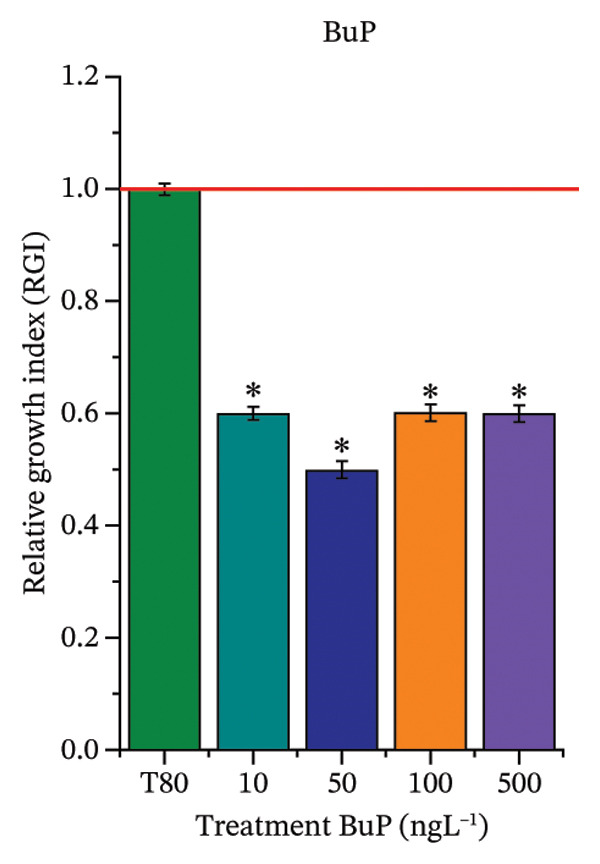
(k)

**Figure:**
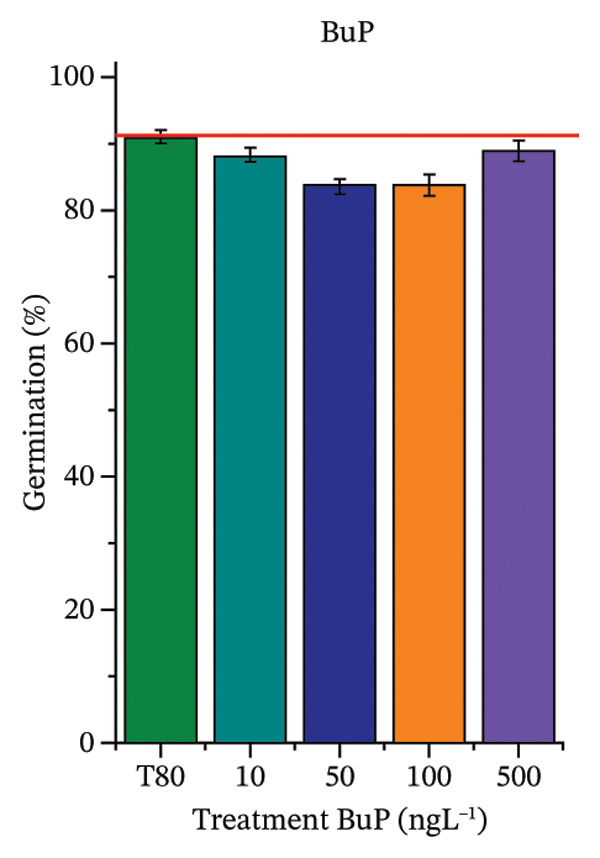
(l)

**Figure:**
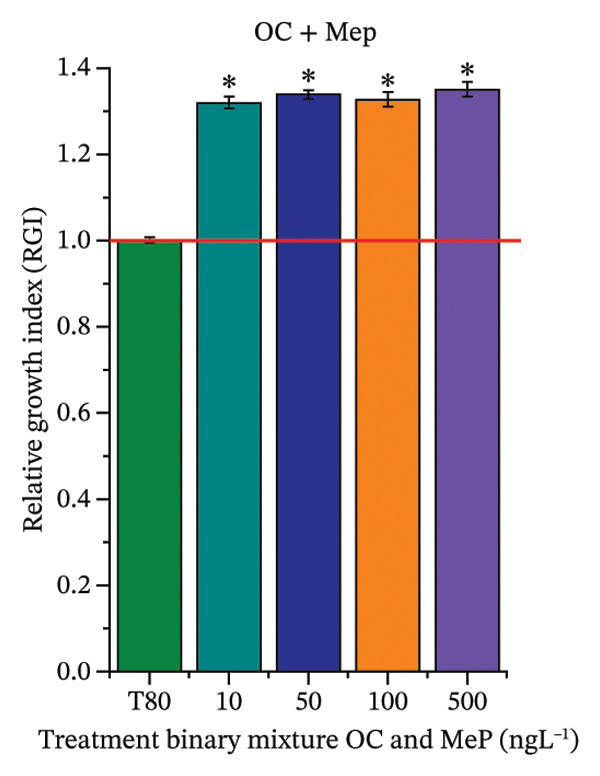
(m)

**Figure:**
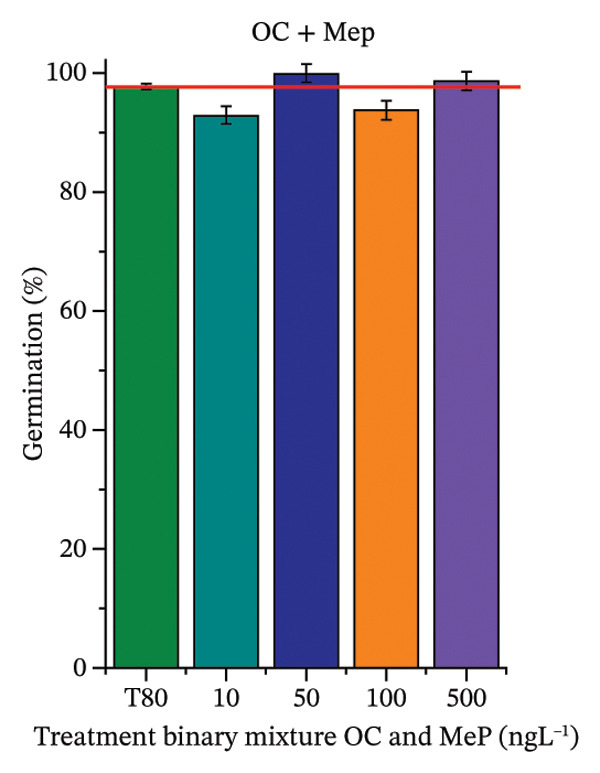
(n)

**Figure:**
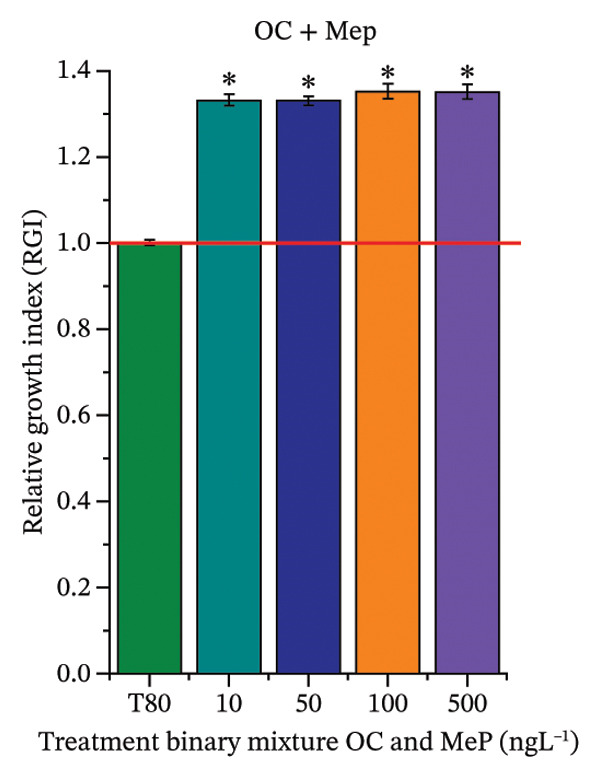
(o)

**Figure:**
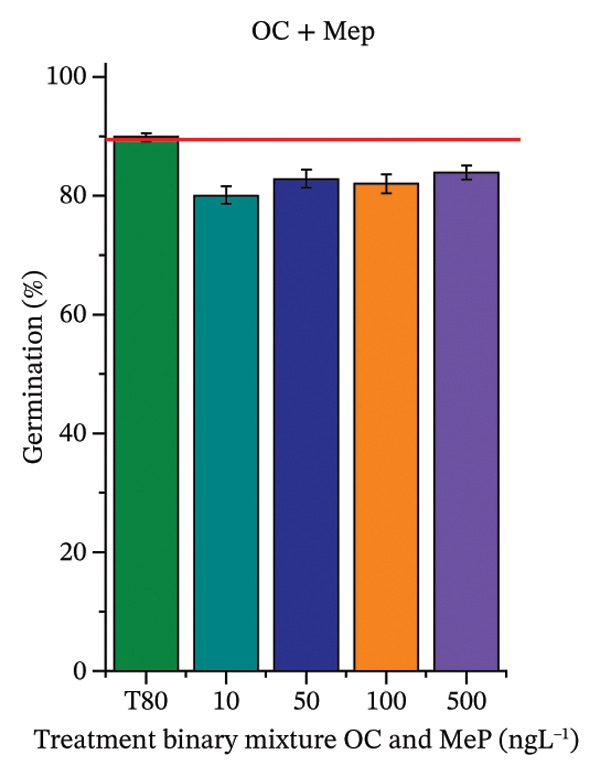
(p)

**Figure:**
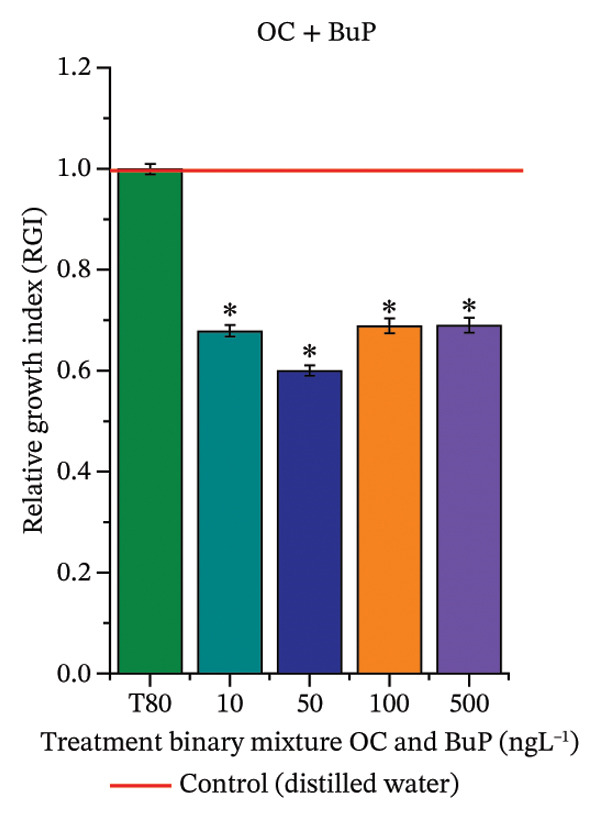
(q)

**Figure:**
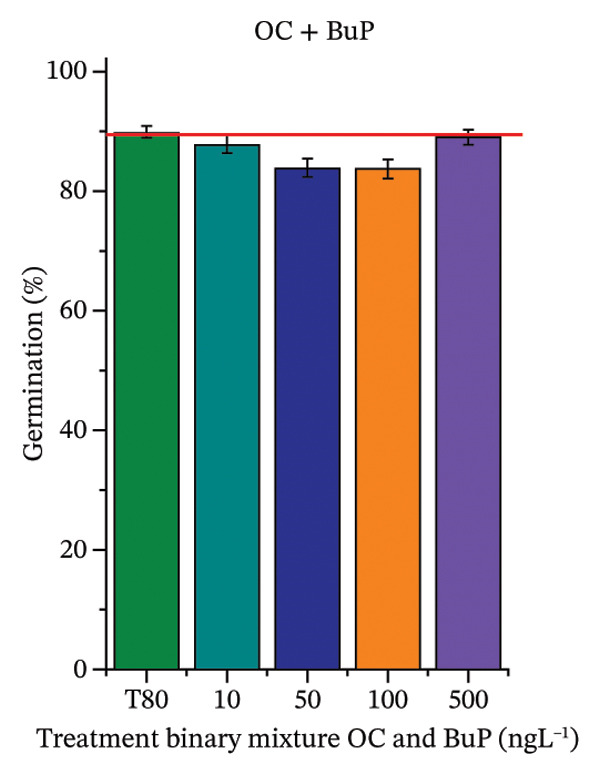
(r)

**Figure:**
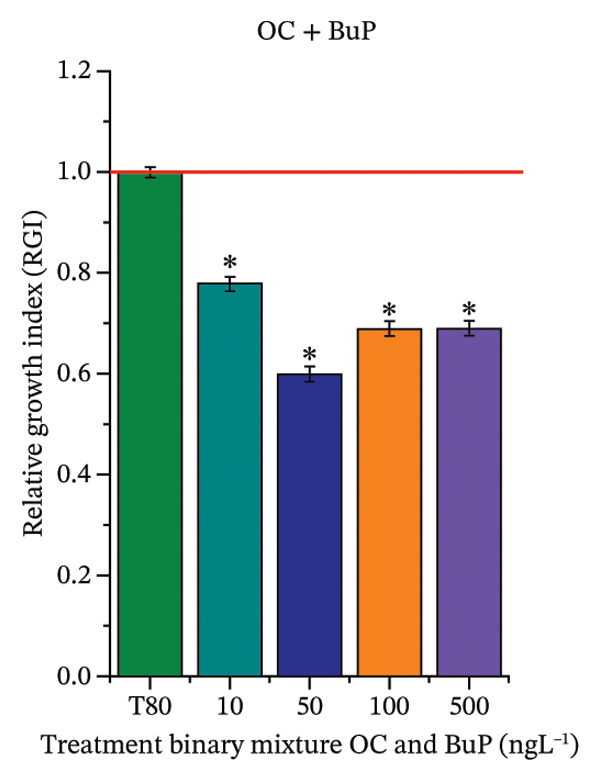
(s)

**Figure:**
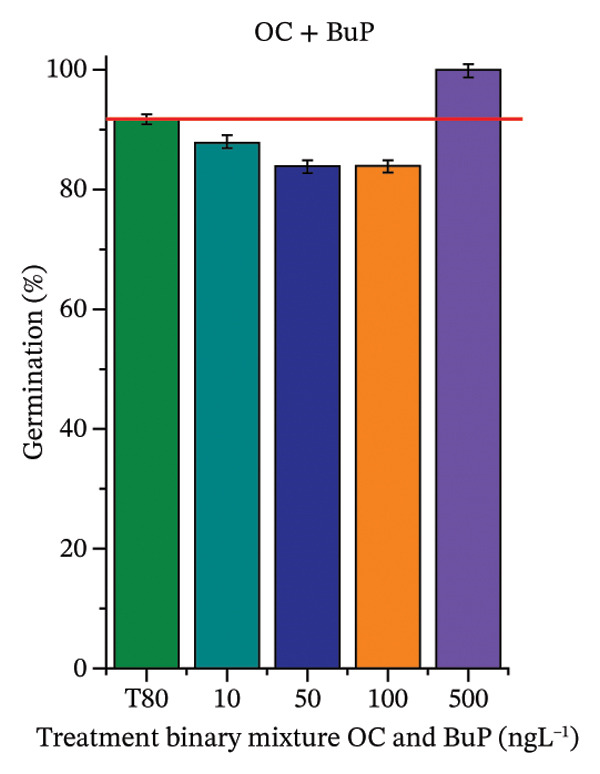
(t)

In Figure 2, the OC + MeP combination did not interfere with germination (Figures 2(m) and 2(p)) and stimulated root growth in *C*. *sativus* and *L*. *esculentum* (RGI > 1.2) (Figures 2(m) and 2(o)). However, at all evaluated concentrations of this mixture, the roots formed in both species exhibited reduced structural integrity compared to the control, breaking easily upon minimal contact. The OC + BuP combination did not affect germination (Figures 2(r) and 2(t)) but promoted a reduction in radicle length in both species at all evaluated concentrations (RGI < 0.8) (Figures 2(q) and 2(s)). In cucumber and tomato, no alterations were observed in root color, morphology, or structural integrity following exposure to OC + BuP, nor were changes observed in root morphology and color in plants exposed to OC + MeP.

In Figure 3, based on root growth in *A*. *cepa* bulbs, only BuP significantly reduced root length (Figure 3(g)). In mixture, OC + MeP stimulated root growth (Figure 3(j)), reaching values up to 59.9% higher than the control (at 500 ng·L^−1^); however, the roots obtained at all evaluated concentrations exhibited reduced structural integrity, fragmenting easily upon minimal contact compared to those grown in distilled water. The OC + BuP mixture, in turn, significantly reduced root growth in bulbs at all concentrations evaluated (Figure 3(m)). Exposure of bulbs to OC, MeP, and BuP, alone or in mixture, did not cause changes in root coloration or morphology.

FIGURE 3Phytotoxicity, cytotoxicity, and genotoxicity of octocrylene, methylparaben, and butylparaben alone, and of octocrylene in binary mixtures with methylparaben or butylparaben, in *Allium cepa* L. roots. Roots were exposed to concentrations of 10, 50, 100, and 500 ng·L^−1^. Effects were evaluated using the parameters average root length (ARL), Mitotic Index (MI), and Cell Alteration Index (CAI). ^∗^Significant difference in relation to controls and ^#^significant difference between binary mixtures and their respective isolated micropollutants, according to the Kruskal–Wallis H test, followed by Dunn’s post hoc test (*p* ≤ 0.05). OC, octocrylene; MeP, methylparaben; BuP, butylparaben; T80, Tween 80 (500 ng·L^−1^); MMS, methylmethanesulfonate (10 µg·L^−1^). (a) ARL obtained for OC (10, 50, 100, and 500 ng·L^−1^); (b) MI (%) obtained for OC 10, 50, 100, and 500 ng·L^−1^); (c) CAI obtained for OC (10, 50, 100, and 500 ng·L^−1^); (d) ARL obtained for MeP (10, 50, 100, and 500 ng·L^−1^); (e) MI (%) obtained for MeP (10, 50, 100, and 500 ng·L^−1^); (f) CAI obtained for MeP (10, 50, 100, and 500 ng·L^−1^); (g) ARL obtained for BuP (10, 50, 100, and 500 ng·L^−1^); (h) MI (%) obtained for BuP (10, 50, 100, and 500 ng·L^−1^); (i) CAI obtained for BuP (10, 50, 100, and 500 ng·L^−1^); (j) ARL obtained for OC + MeP (10, 50, 100, and 500 ng·L^−1^); (k) MI (%) obtained for OC + MeP 10, 50, 100, and 500 ng·L^−1^); (l) CAI obtained for OC + MeP (10, 50, 100, and 500 ng·L^−1^); (m) ARL obtained for OC + BuP (10, 50, 100, and 500 ng·L^−1^); (n) MI (%) obtained for OC + BuP (10, 50, 100, and 500 ng·L^−1^); and (o) CAI obtained for OC + BuP (10, 50, 100, and 500 ng·L^−1^).

**Figure:**
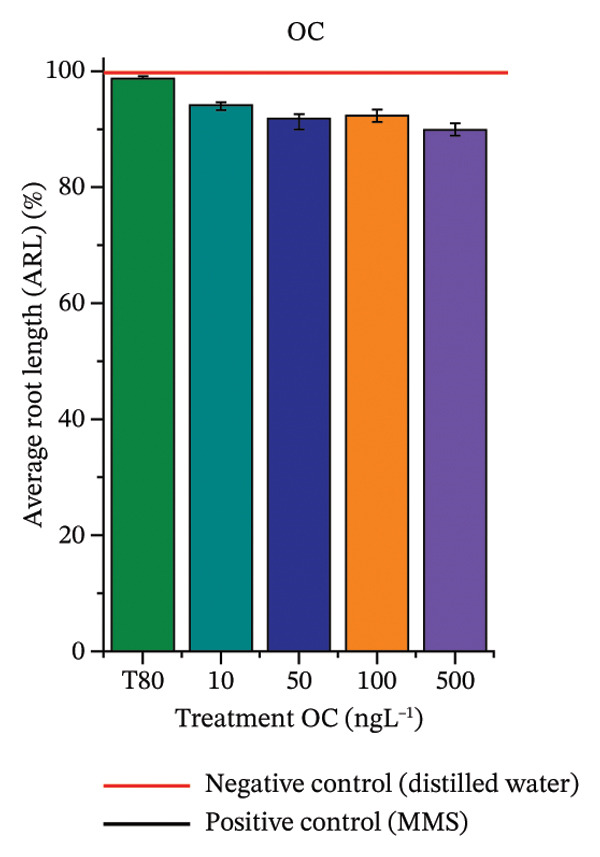
(a)

**Figure:**
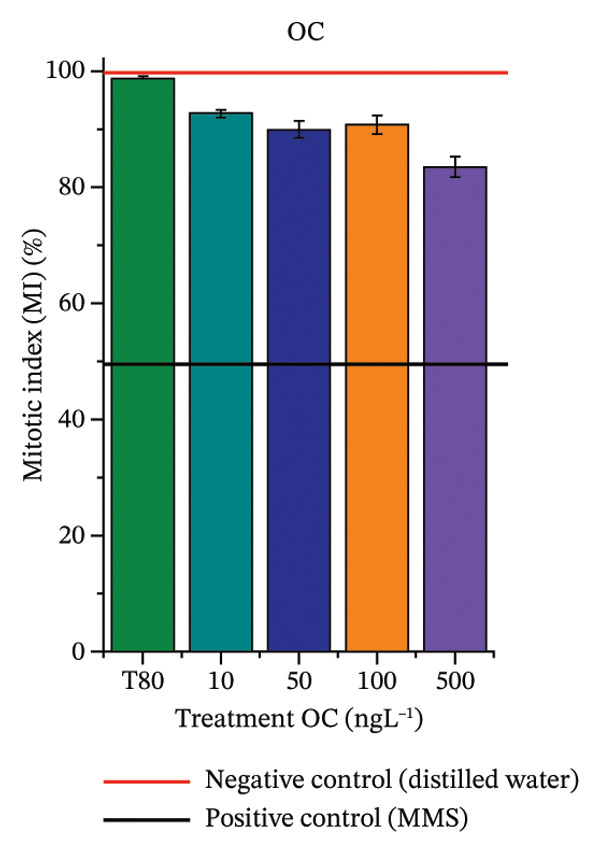
(b)

**Figure:**
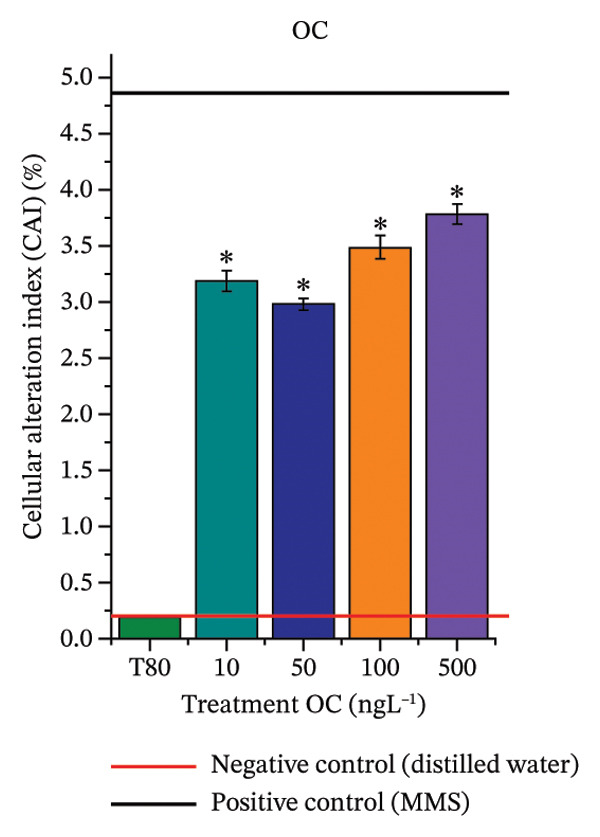
(c)

**Figure:**
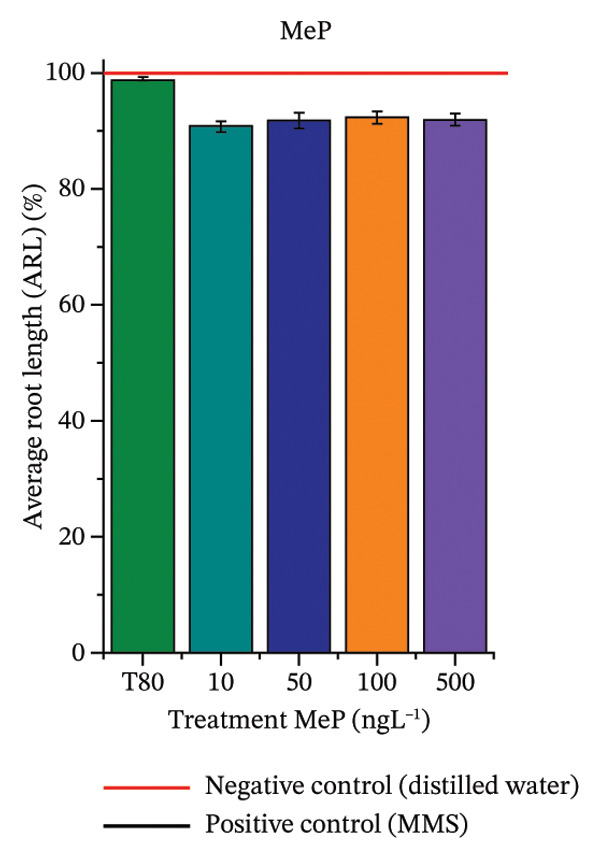
(d)

**Figure:**
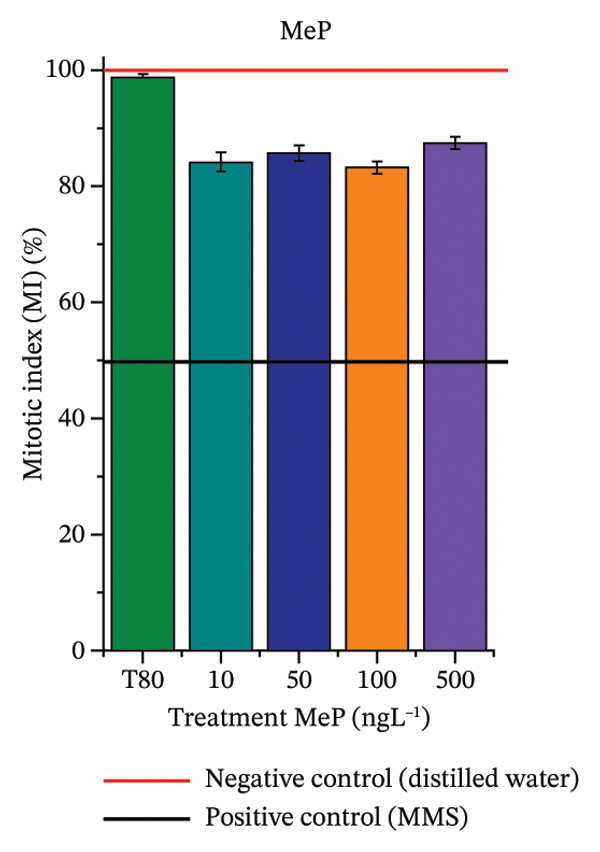
(e)

**Figure:**
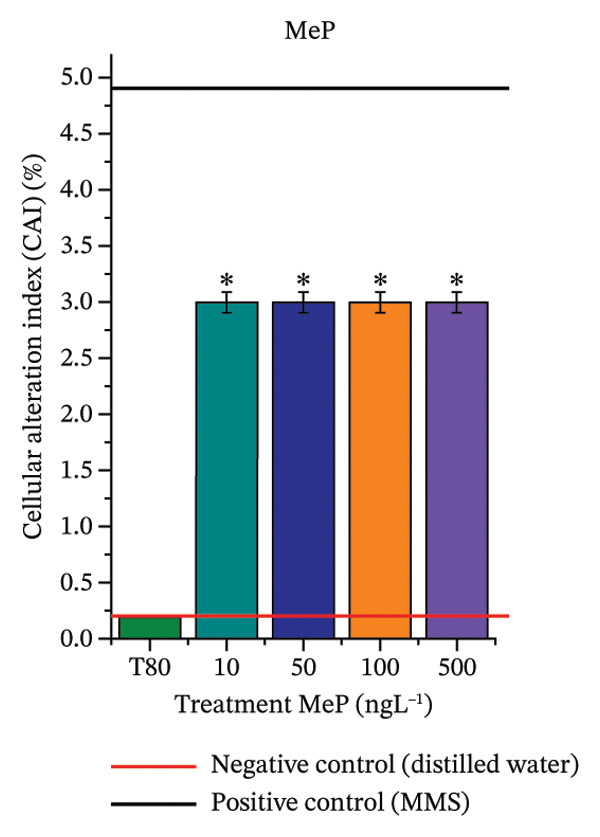
(f)

**Figure:**
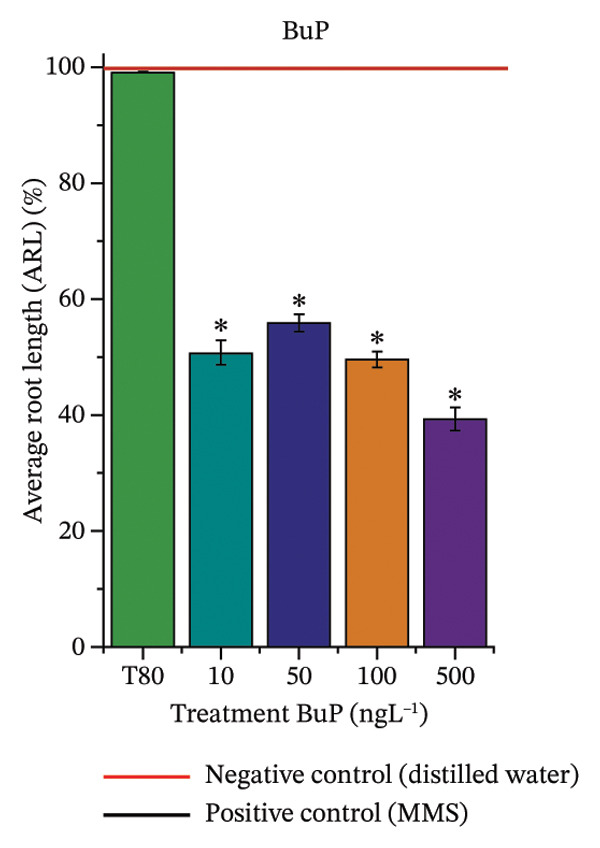
(g)

**Figure:**
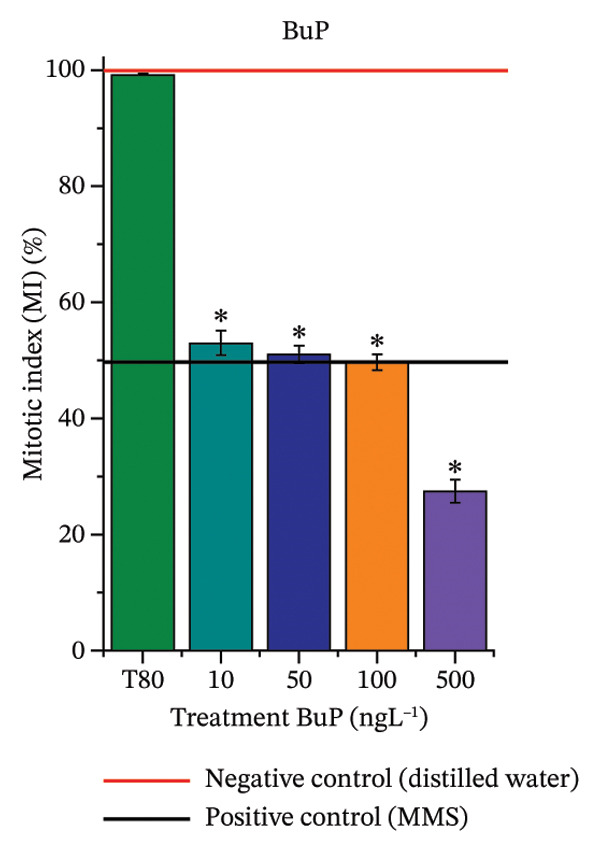
(h)

**Figure:**
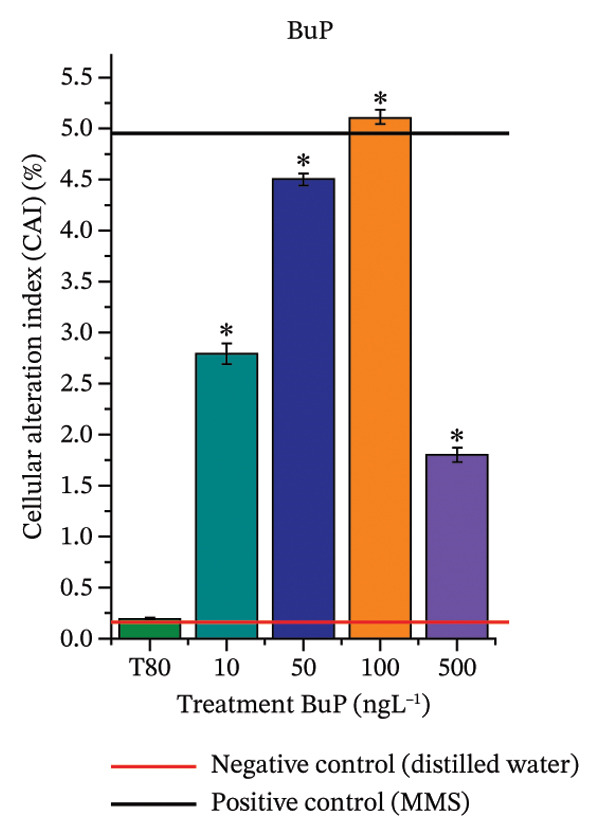
(i)

**Figure:**
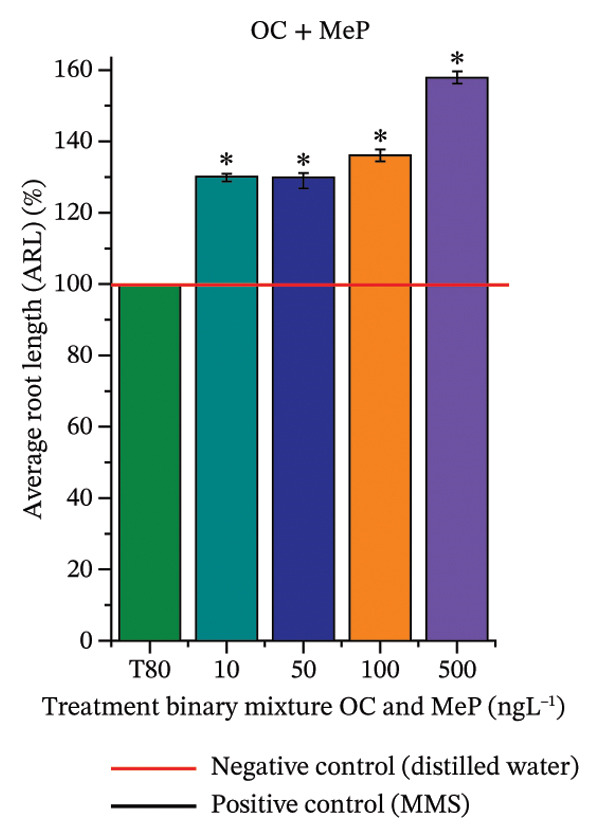
(j)

**Figure:**
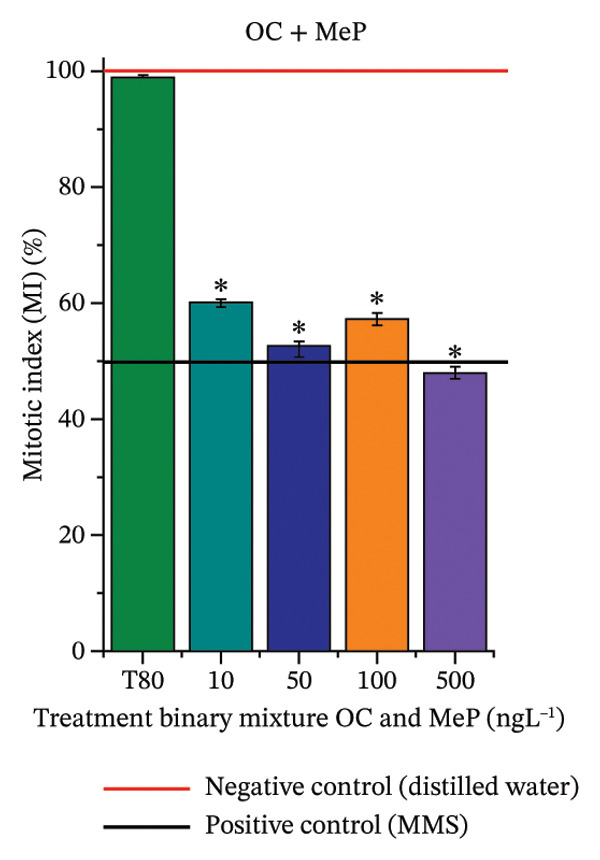
(k)

**Figure:**
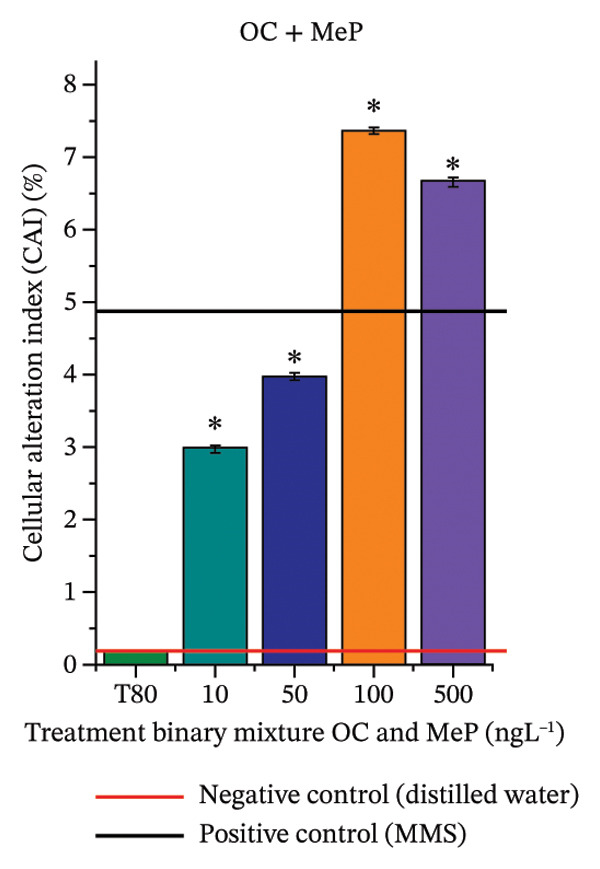
(l)

**Figure:**
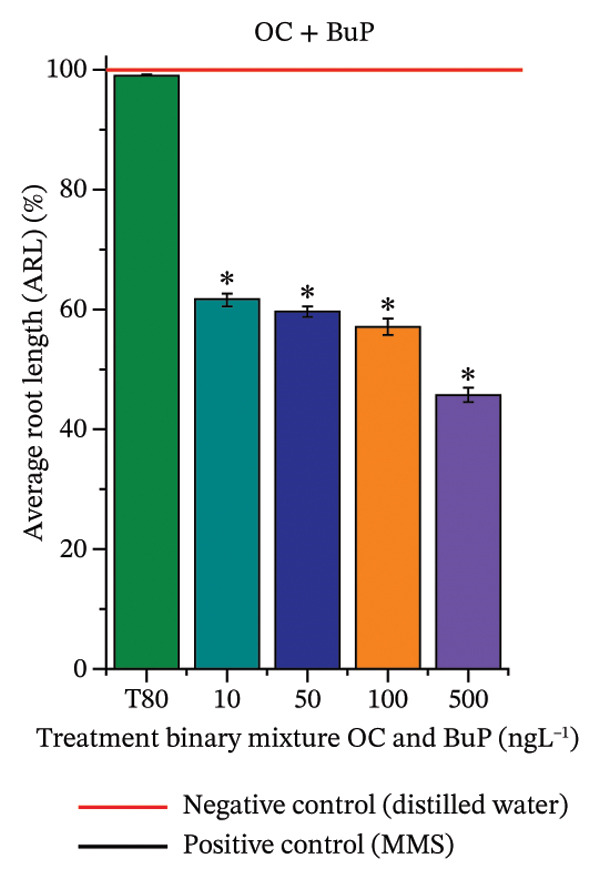
(m)

**Figure:**
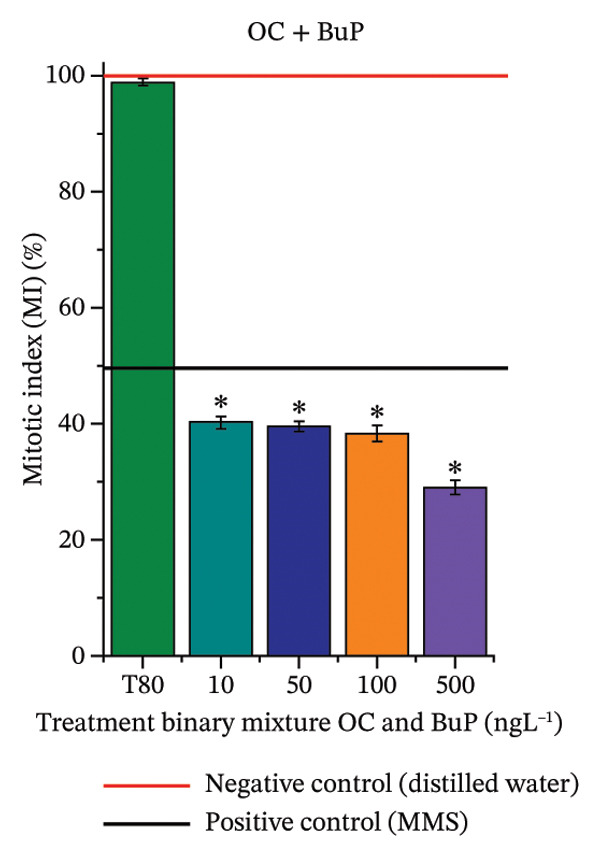
(n)

**Figure:**
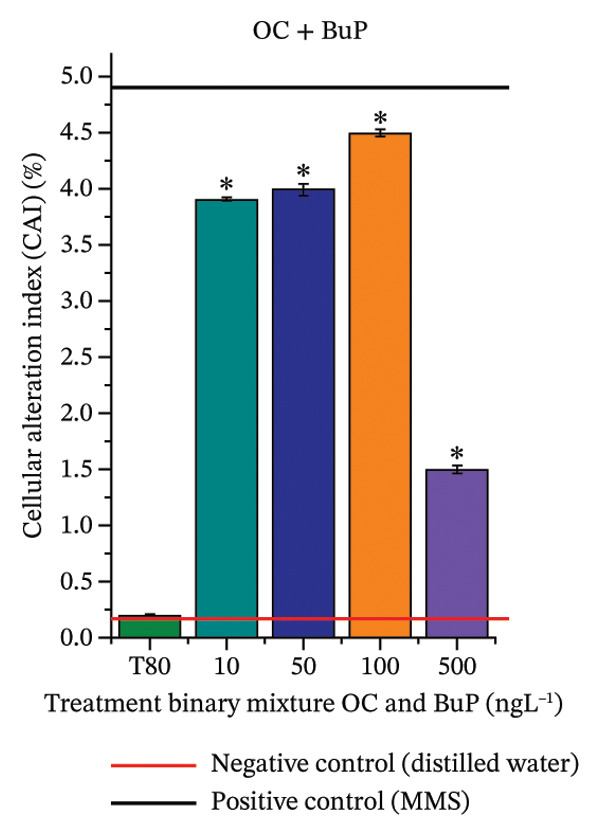
(o)

### 3.3. Cytotoxicity and Aneugenic and Clastogenic Potentials in Roots of *A. cepa*

In Figure 3, OC and MeP alone did not cause a significant reduction in cell proliferation in the root meristems of *A. cepa* (Figures 3(b) and 3(e)), unlike BuP (Figure 3(h)). The OC + MeP and OC + BuP mixtures induced a significant mitodepressive effect in the apical region of onion roots (Figures 3(k) and 3(n)), with the OC + BuP mixture showing the greatest effect, resulting in cell division rates below 50% (Figure 3(n)) and demonstrating severe cytotoxicity to meristems.

**FIGURE 4:**
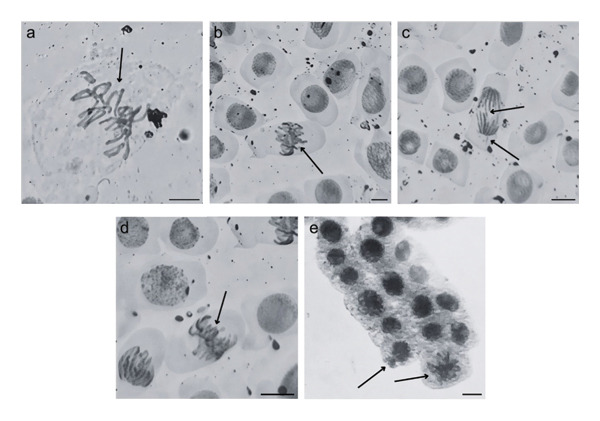
Cellular changes observed in root meristems of *Allium cepa* L. bulbs treated with octocrylene, methylparaben, and butylparaben alone, and octocrylene in a binary mixture with methylparaben and butylparaben, at concentrations of 10, 50, 100, and 500 ng·L^−1^. (a) Chromosomal disorganization in metaphase with chromosome loss, (b) chromosomal disorganization in metaphase, (c) anaphase bridge and chromosome loss, (d) sticky metaphase, and (e) micronuclei and chromosomal disorganization in prophase. Scale bar: 10 µm.

Based on Figures 3 and 4, BuP and the mixtures OC + MeP and OC + BuP induced cellular changes (Figures 3(l) and 3(o)), demonstrating a significant genotoxic effect on the root meristems of *A. cepa*. BuP promoted mitotic spindle alterations characterized by chromosomal disorganization in metaphase (Figures 4(a) and 4(b)). OC + MeP also caused mitotic spindle alterations, evidenced by chromosomal disorganization in metaphase, accompanied by chromosomal loss (Figure 4(a)); chromosomal disorganization in metaphase (Figure 4(b)); anaphase bridge and chromosome loss (Figure 4(c)), and micronucleus and disorganization in prophase (Figure 4(e)). OC + BuP, in addition to these alterations, induced metaphases with adhered (sticky) chromosomes (Figure 4(d)).

Furthermore, OC + MeP and OC + BuP induced the formation of micronuclei (Figure 4(e)) in root meristems, all concentrations being evaluated. It is noteworthy that at the highest concentration evaluated (500 ng·L^−1^), OC + BuP caused the lowest frequency of cellular alterations among the concentrations evaluated (Figure 3(o)), a condition due to the severe mitodepressive effect caused by this concentration on bulb root meristems, which resulted in MI below 40% (Figure 3(n)). Therefore, according to Figures 3 and 4, in bulb root meristems, BuP, OC + MeP, and OC + BuP, at the four concentrations evaluated, triggered genotoxicity with high aneugenic potential, as evidenced by disturbances to the mitotic spindle, and high clastogenic potential, as evidenced by the high frequency of micronuclei.

### 3.4. Oxidative Stress in Root Meristems of *A. cepa* Subjected to Mixtures

In Figure 5, the results of the DPPH assay (Figure 5(a)) indicate that OC + MeP concentrations compromised the antioxidant system in bulb root meristems, as evidenced by the reduced total antioxidant capacity and further supported by decreased phenolic compound concentrations (Figure 5(c)). This condition corroborates the modulation of antioxidant enzymes observed for this mixture at all concentrations evaluated (Figure 6), in which catalase (CAT) activity was inhibited (Figure 6(a)) and ascorbate peroxidase (APX) activity was significantly increased (Figure 6(b)), indicating H_2_O_2_ accumulation in the meristems. Furthermore, the increase in SOD activity (Figure 6(d)) at concentrations of 10, 50, and 100 ng·L^−1^ indicates an increase in the formation of superoxide anion in the cells; however, at 500 ng·L^−1^, this enzyme was significantly inhibited, demonstrating the accumulation of these reactive species in the meristems. In summary, the results indicate that exposure to OC + MeP concentrations promoted an imbalance in the redox homeostasis of *A. cepa* root meristems, mainly due to the accumulation of H_2_O_2_.

FIGURE 5Lipid peroxidation (TBARS) and phenolic compound concentration (FC) in roots of *Allium cepa* L. bulbs exposed to octocrylene in binary mixtures with methylparaben or butylparaben. Bulbs were exposed to concentrations of 10, 50, 100, and 500 ng·L^−1^. ^∗^Significant difference compared to controls, according to the Kruskal–Wallis H test, followed by Dunn’s post hoc test (*p* ≤ 0.05). OC, octocrylene; MeP, methylparaben; BuP, butylparaben; T80, Tween 80 (500 ng·L^−1^). (a) DPPH obtained for OC + MeP (10, 50, 100, and 500 ng·L^−1^); (b) TBARS obtained for OC + MeP (10, 50, 100, and 500 ng·L^−1^); (c) FC obtained for OC + MeP (10, 50, 100, and 500 ng·L^−1^); (d) DPPH obtained for OC + BuP (10, 50, 100, and 500 ng·L^−1^); (e) TBARS obtained for OC + BuP (10, 50, 100, and 500 ng·L^−1^); and (f) FC obtained for OC + BuP (10, 50, 100, and 500 ng·L^−1^).

**Figure:**
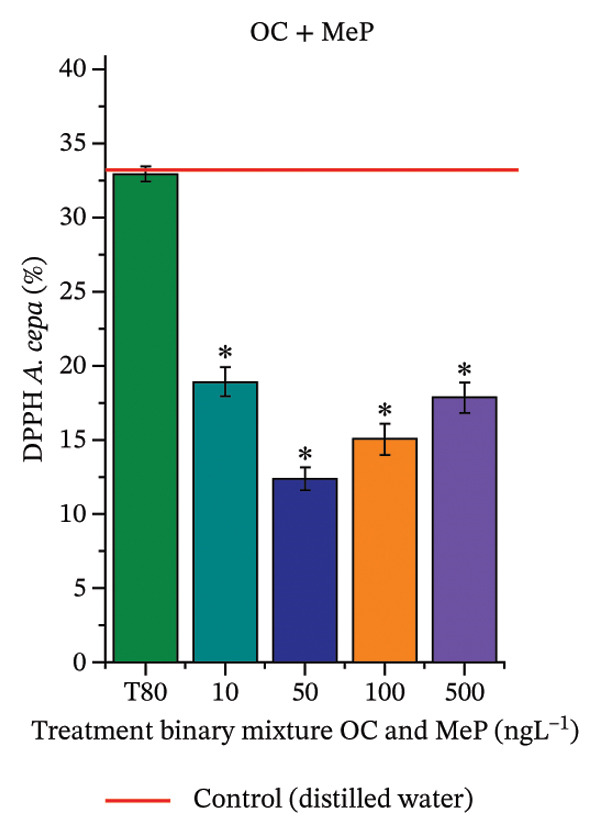
(a)

**Figure:**
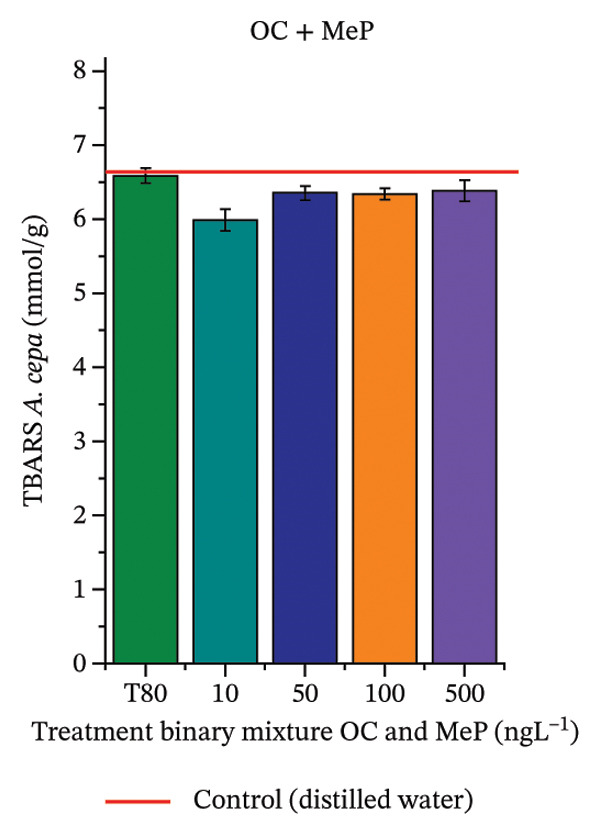
(b)

**Figure:**
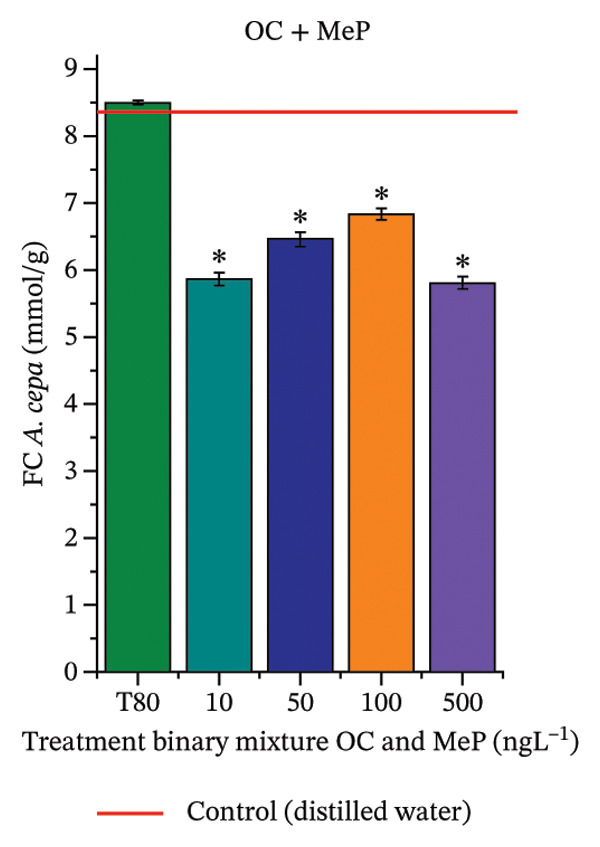
(c)

**Figure:**
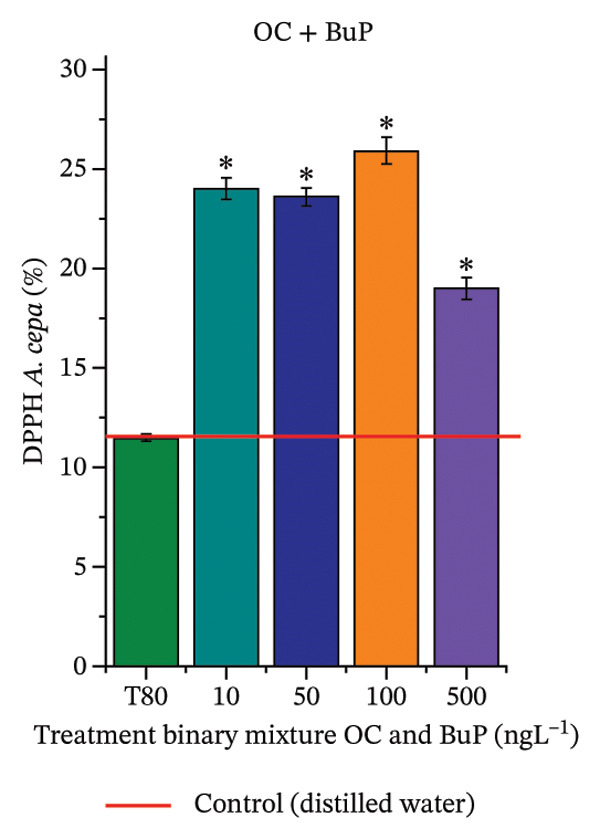
(d)

**Figure:**
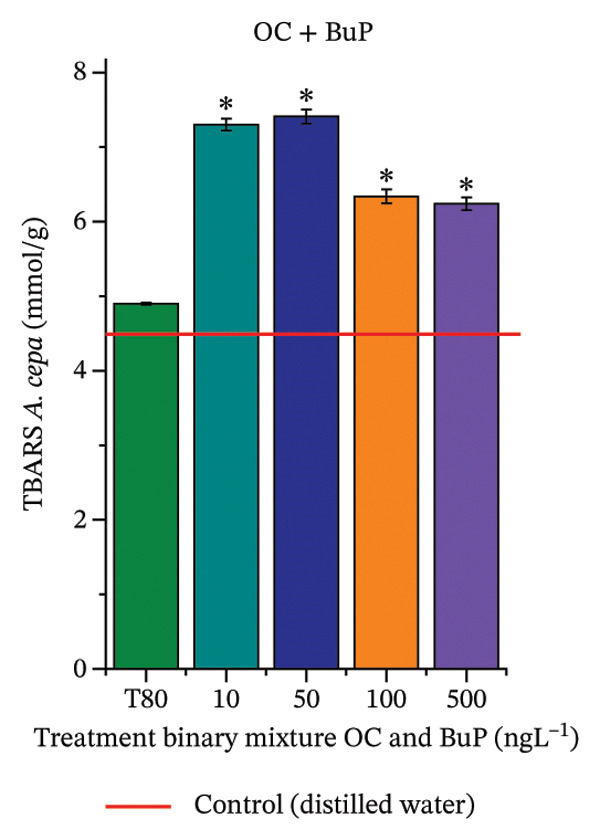
(e)

**Figure:**
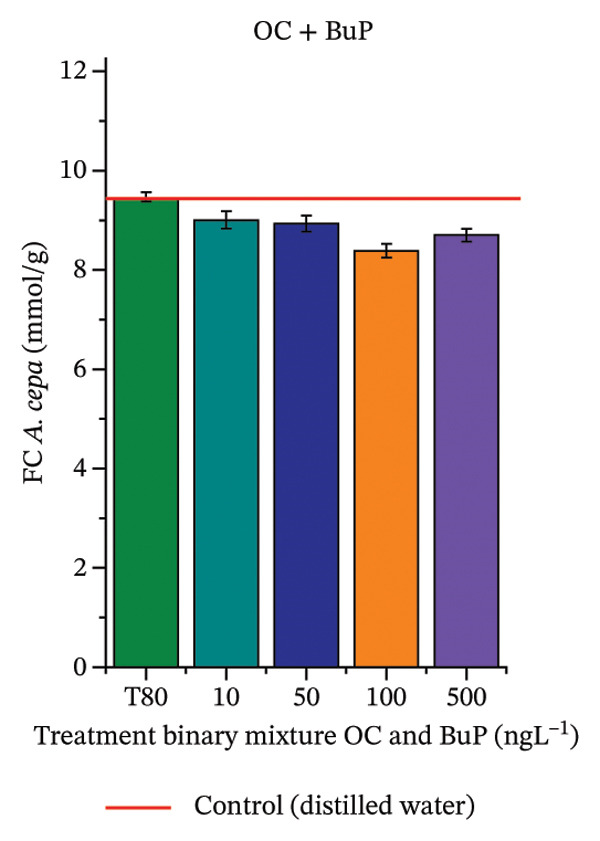
(f)

FIGURE 6Activity of the enzymes catalase (CAT), ascorbate peroxidase (APX), guaiacol peroxidase (GPOX), and superoxide dismutase (SOD) in meristems of *Allium cepa* L. bulb roots exposed to octocrylene in binary mixtures with methylparaben or butylparaben. Bulbs were exposed to concentrations of 10, 50, 100, and 500 ng·L^−1^. ^∗^Significant difference about the controls according to Kruskal–Wallis H, followed by Dunn’s post hoc test (*p* ≤ 0.05). OC, octocrylene; MeP, methylparaben; BuP, butylparaben; T80, Tween 80 (500 ng·L^−1^). (a) CAT obtained for OC + MeP (10, 50, 100, and 500 ng·L^−1^); (b) APX obtained for OC + MeP (10, 50, 100, and 500 ng·L^−1^); (c) GPOX obtained for OC + MeP (10, 50, 100, and 500 ng·L^−1^); (d) SOD obtained for OC + MeP (10, 50, 100, and 500 ng·L^−1^); (e) CAT obtained for OC + BuP (10, 50, 100, and 500 ng·L^−1^); (f) APX obtained for OC + BuP (10, 50, 100, and 500 ng·L^−1^); (g) GPOX obtained for OC + BuP (10, 50, 100, and 500 ng·L^−1^); and (h) SOD obtained for OC + BuP (10, 50, 100, and 500 ng·L^−1^).

**Figure:**
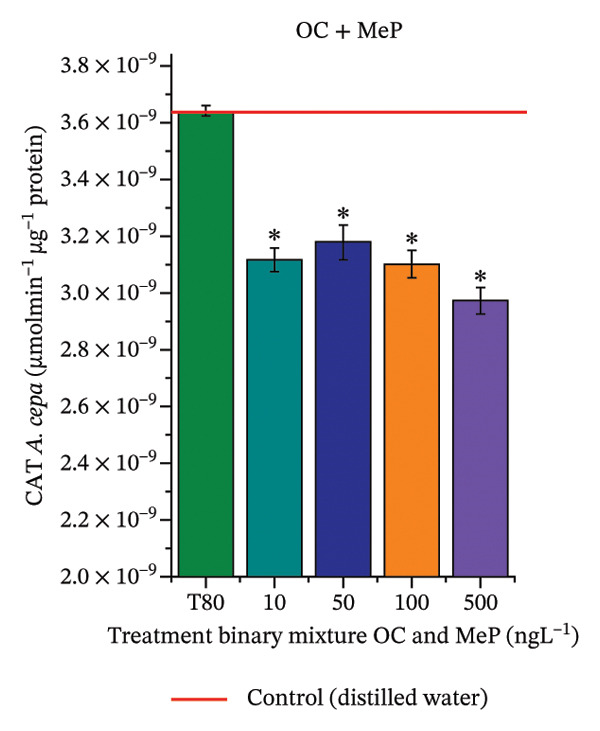
(a)

**Figure:**
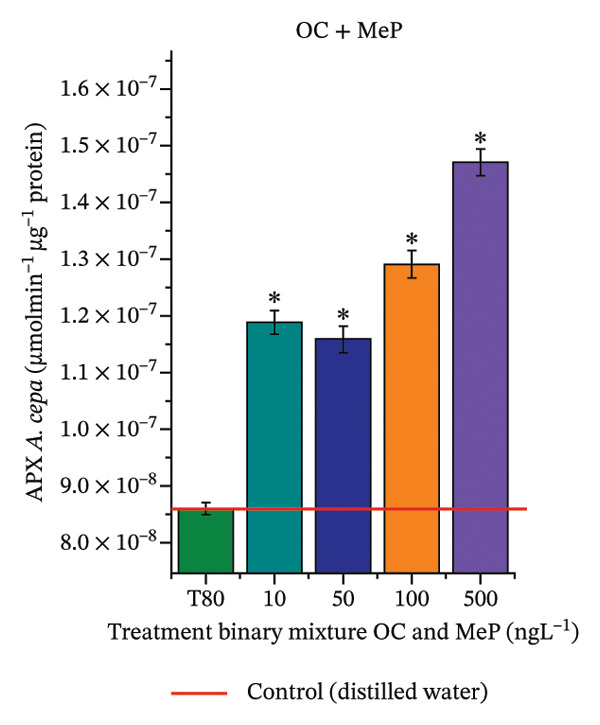
(b)

**Figure:**
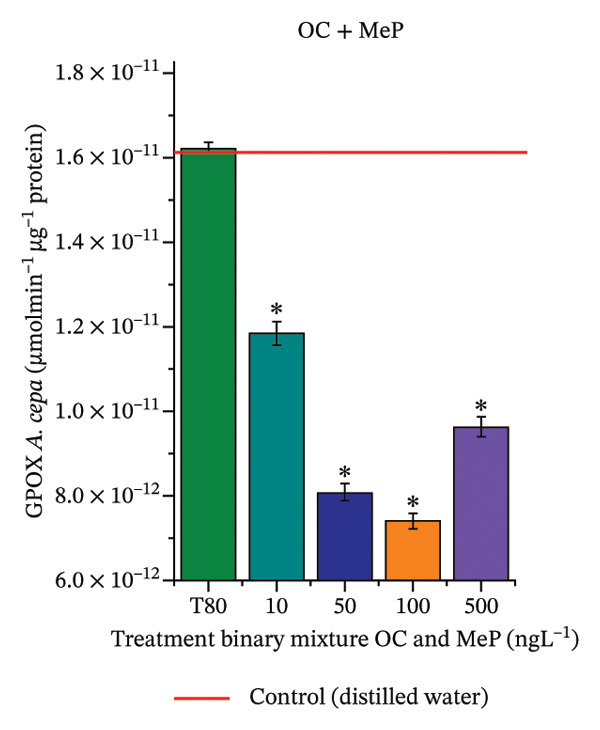
(c)

**Figure:**
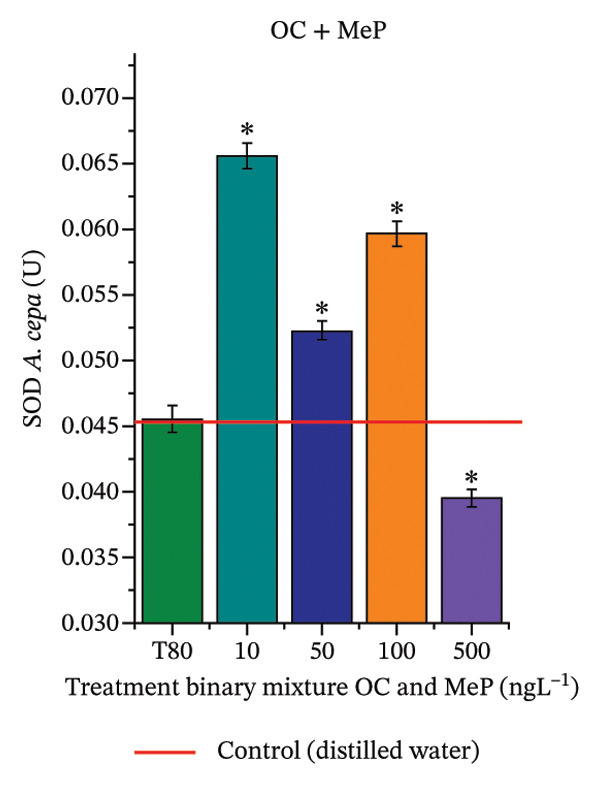
(d)

**Figure:**
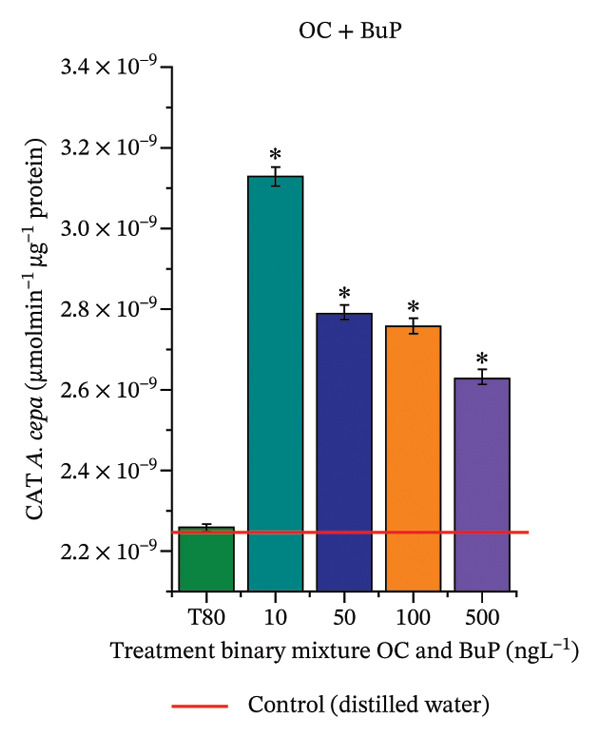
(e)

**Figure:**
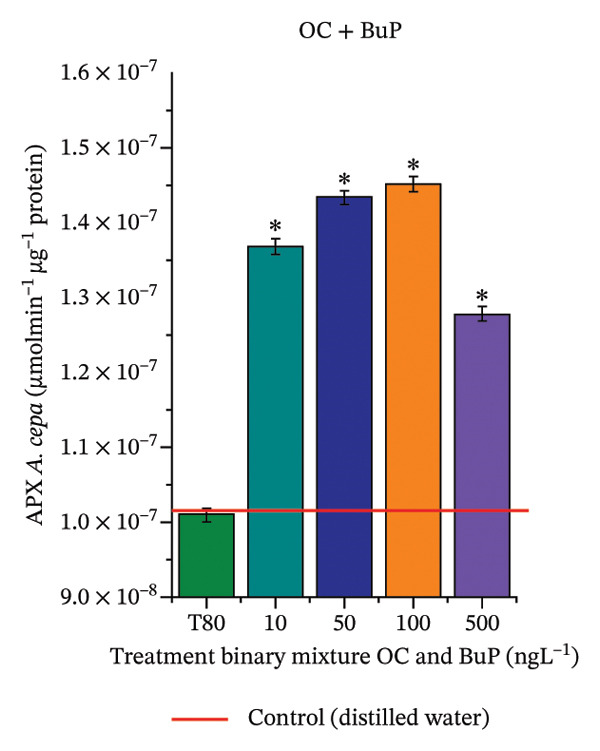
(f)

**Figure:**
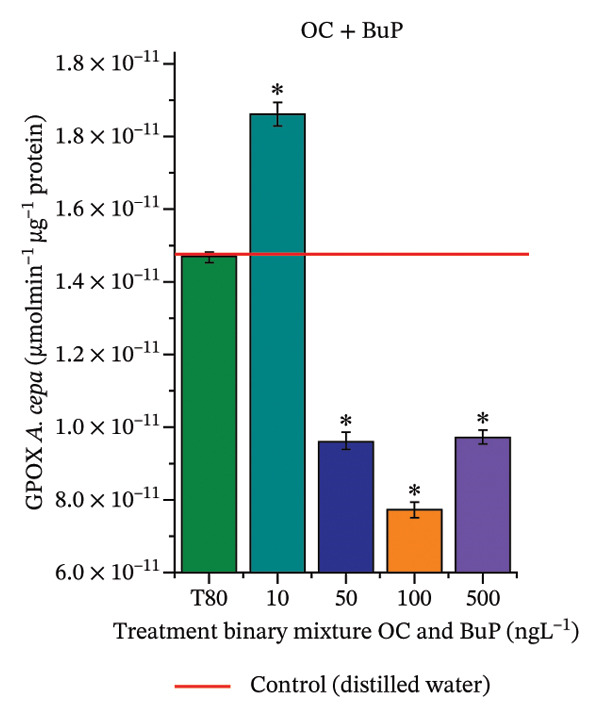
(g)

**Figure:**
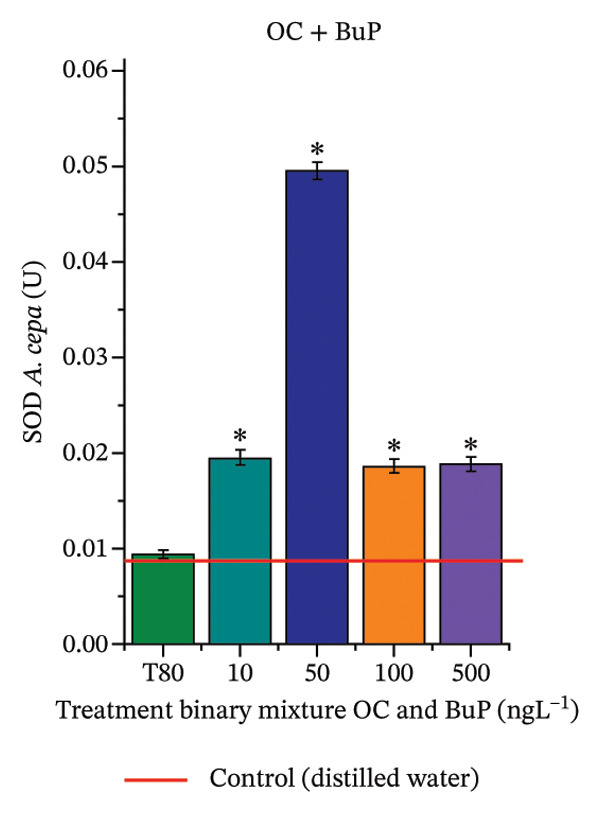
(h)

In Figure 5, the DPPH results show that the OC + BuP mixtures promoted a significant increase in total antioxidant capacity (Figure 5(d)), indicating activation of the cellular antioxidant system. However, the concentration of phenolic compounds did not increase (Figure 5(f)). In addition, significant increases in CAT, APX, and SOD activities were observed (Figures 6(e), 6(f) and 6(h)), indicating coordinated activity among these enzymes. These results suggest that although exposure to the OC + BuP mixture increased H_2_O_2_ levels, *A. cepa* root meristems efficiently modulated oxidative stress, maintaining cellular redox homeostasis.

For OC + MeP, no changes in TBARS levels were observed (Figure 5(b)). However, GPOX activity was significantly reduced (Figure 6(c)), suggesting that compensatory antioxidant mechanisms controlled lipid peroxidation in membranes. In contrast, OC + BuP promoted lipid peroxidation in root meristems, evidenced by a significant increase in TBARS levels (Figure 5(e)). For this mixture, GPOX activity increased significantly at a concentration of 10 ng·L^−1^ (Figure 6(g)), indicating an initial antioxidant response to the oxidizing agents during lipid peroxidation but insufficient to prevent oxidative damage. At concentrations of 50, 100, and 500 ng·L^−1^ of OC + BuP, GPOX activity was significantly inhibited (Figure 6(g)) due to the intensification of lipid oxidation, which caused the formation of highly oxidizing reactive species and, consequently, worsening cellular oxidative stress.

## 4. Discussion

### 4.1. Differential Activation of Oxidative Pathways and Common Toxic Effects in Root Meristems

In meristematic tissues, the antioxidant enzymes CAT, APX, and SOD are essential for maintaining redox balance and for proper cell cycle regulation [11, 67]. In the present study, exposure to the OC + MeP mixture dysregulated the activity of these enzymes in the root apical region, compromising the antioxidant system’s efficiency and probably leading to H_2_O_2_ accumulation in the meristems (Figures 5(a), 5(c), 6(a), 6(b), 6(d)). In contrast, the OC + BuP mixture, although not causing a detectable imbalance in redox homeostasis (Figures 6(e), 6(f) and 6(h)), induced lipid peroxidation in the roots, a condition that, in addition to disrupting cellular membranes, leads to the formation of highly reactive species such as hydroxyl, hydroperoxyl, aldehydes, and ketone radicals.

Excess H_2_O_2_ and lipid oxidation products are reactive species with high oxidative potential in root meristems [68]. During interphase, these reactive agents have the potential to oxidize proteins involved in DNA replication, protein synthesis, and cell cycle checkpoint regulation, compromising cell duplication and favoring the interruption or delay of cell cycle progression, leading to a consequent reduction in cell proliferation in meristematic tissues [68, 69]. Furthermore, during mitosis, these reactive species can affect structural proteins of chromatin and the mitotic spindle, altering chromosome compaction, microtubule stability, and correct chromosome segregation, which can result in chromosomal disorganization, chromosome loss, delayed chromosomes, micronuclei, and adherent chromosomes [70, 71].

Thus, although OC + MeP and OC + BuP triggered oxidative stress through different pathways—imbalance of redox homeostasis and lipid peroxidation, respectively (Figures 5 and 6)—both mixtures converged on the same toxic outcome in root meristems, evidenced by significant cytotoxicity and genotoxicity at all concentrations evaluated for these mixtures (Figures 3(k) and 3(n)).

### 4.2. Cytogenetic Disorders, Root Growth, and Interactions Between Mixed Micropollutants

The significant reduction in cell proliferation observed in the root meristems of *A. cepa* at all evaluated concentrations of OC + MeP and OC + BuP (Figures 3(k) and 3(n)) demonstrates the high cytotoxic potential of these mixtures, associated with disturbances in cell cycle progression during interphase. Among the mixtures, OC + BuP presented the highest degree of cytotoxicity to the roots (Figure 3(n)), since all evaluated concentrations resulted in mitotic indices below 50%, a condition that, according to Mohammed et al. [72], characterizes severe toxicity with potential loss of the proliferative capacity of root meristems.

Furthermore, OC + MeP and OC + BuP demonstrated high aneugenic potential (Figure 4), in which the disorganization, delay, and chromosomal losses observed (Figures 4(a), 4(b), 4(c) and 4(d)) imply chromosomal imbalance and may result, after successive cell cycles, in aneuploid and polyploid cells [47]. In meristems, disturbances in mitotic checkpoints and cells with different chromosome numbers can disrupt the expression of essential genes and cause subsequent metabolic changes, severely impairing the development of secondary tissues in plants [73, 74]. Cells with gene imbalance can be selectively eliminated from young tissues when their functioning is unviable; however, this condition is also harmful to the plant because it further enhances cytotoxicity in meristems [48, 75].

In addition to chromosomal disorganization (Figures 4(a), 4(b) and 4(c)), OC + BuP significantly induced the formation of sticky chromosomes in metaphase (Figure 4(e)), which are alterations resulting from abnormal adhesion between chromatin and/or chromosomes due to changes in the structural organization of chromatin. This type of abnormality can compromise proper chromosomal segregation, favoring the formation of bridges, breaks, and chromosome loss, and is classified as a serious form of cellular damage in plants because it is often associated with the functional loss of meristems [76, 77].

Furthermore, OC + MeP and OC + BuP induced the formation of micronuclei (Figure 4(e)) in root meristems, demonstrating significant interference by these mixtures in chromosome segregation during cell division. According to Leme and Marin‐Morales [47], the high frequency of micronuclei characterizes persistent genomic instability, with a high potential to compromise proliferative activity in young tissues and the structural and functional development of secondary tissues in plants.

The phytotoxicity results observed for radicles of *C. sativus* and *L. esculentum* (Figures 2(q) and 2(s)) and for roots of *A. cepa* bulbs (Figure 3(m)), subjected to OC + BuP concentrations, corroborate the cytotoxicity and genotoxicity observed for the root meristems of *A. cepa* (Figures 3(n) and 3(o)). However, the significant mitodepressive effect induced by OC + MeP in onion root meristems (Figure 3(k)) contrasts with the effects observed in cucumber and tomato radicles, in which exposure to this mixture, at all concentrations evaluated, promoted root growth stimulation (RGI > 1.2) (Figures 2(m) and 2(o)), as well as the effects observed in bulbs, in which concentrations of this mixture formed roots with lengths significantly greater than the control roots (Figure 3(j)).

It should be noted that root growth is regulated in meristems by cell division and cell elongation, which are physiologically independent processes [45]. Based on this context, it can be inferred that, although OC + MeP caused a significant reduction in cell division at the root tips (Figure 3(j)), the radicles of *C. sativus* and *L. esculentum*, as well as the roots of *A. cepa* bulbs, continued to grow through the elongation of preexisting cells, without associated cell proliferation. According to Shishkova et al. [78], this mechanism constitutes a temporary defensive response in plants to stressors, as the number of cells does not increase. The potential for cell elongation is physiologically limited and may result in longer roots with lower resistance to mechanical stimuli—conditions observed in this study for onion, cucumber, and tomato roots, respectively.

Similar conditions were observed by Herrero et al. [45], who evaluated the effects of the micropollutant triclosan (in concentrations in mg·L^−1^) on *A. cepa* roots and found that, although significant root elongation occurred, cytotoxicity results demonstrated that the tested concentrations of this antimicrobial caused significant inhibition of cell proliferation and cellular alterations at a significant frequency in onion root meristems.

Regarding the interaction between micropollutants in mixtures, OC and MeP, when evaluated individually, did not induce toxicity at any concentration or in any of the plant species analyzed (Figures 2(a), 2(c), 2(f), and 2(h)). However, although it did not cause apparent phytotoxicity in root growth (Figures 2(m) and 2(o)), the association between these compounds triggered significant cellular toxicity in root meristems (Figures 3(k) and 3(l)), indicating a synergistic interaction between OC and MeP. Moreover, BuP alone induced significant cellular and systemic toxicity in roots (Figures 2(i), 2(k), 3(g), 3(h), and 3(i)). Thus, the toxic effects observed with OC + BuP are similar to those with BuP alone, indicating an additive interaction in which the more toxic compound largely determines the biological response of the mixture.

Only a single study has been found in the literature addressing the ecotoxicity of a sunscreen and paraben mixture, which used an aquatic organism. Vega et al. [15] evaluated nanoparticles of the inorganic UV filter TiO_2_ in a binary mixture with benzylparaben at concentrations in the mg·L^−1^ range and reported a synergistic interaction between the compounds, resulting in high toxicity to *Daphnia magna* L.

### 4.3. Molecular, Cytogenetic, and Root Effects Associated With a Potential Reduction in Plant Physiological Performance

Oxidative stress (Figures 5 and 6), cytotoxicity (Figures 3(k) and 3(n)), and the high aneugenic and clastogenic potential (Figure 4) induced by the OC + MeP and OC + BuP mixtures caused relevant structural and functional changes in the root systems of *C. sativus*, *L. esculentum*, and *A. cepa* (Figures 2 and 3), which may have implications for agronomic performance and productivity of cultivated plants under field conditions. The significant reduction in root growth observed in *C. sativus*, *L. esculentum*, and *A. cepa* exposed to OC + BuP concentrations (Figures 2(q), 2(s), and 3(m)), as well as the formation of mechanically more fragile roots in these plants when subjected to OC + MeP concentrations, suggest that both mixtures have the potential to compromise the functional quality of the root system, albeit through different mechanisms.

Proper root development is crucial for the uptake of water, nutrients, and minerals, as well as for anchorage and tolerance to abiotic stresses such as water deficit and soil compaction [79]. In the present study, the reduction in cell proliferation in root meristems (Figures 3(k) and 3(n)), together with the formation of short or structurally fragile roots observed in *C. sativus*, *L. esculentum*, and *A. cepa* exposed to OC + MeP and OC + BuP (Figure 2; 3(k), and 3(n)), suggests that these mixtures have the potential to negatively affect root system development.

In addition to potential effects on root development, persistent cytogenetic alterations such as aneuploidy and polyploidy, sticky chromosomes, and micronuclei in meristematic tissues—as observed here for *A. cepa* (Figures 4(d) and 4(e))—represent risk factors that may influence crop productivity. According to Hodge et al. [80], Lindsey et al. [81], Kwasniewska and Bara [82] and Lv et al. [83], such cellular alterations can deregulate gene expression and potentially compromise the differentiation and functioning of secondary tissues, resulting in plants that may be less vigorous, metabolically less efficient, and less able to respond to environmental variations.

In the case of the OC + MeP mixture, stimulation of root growth was observed (Figures 2(m), 2(o), 3(j)). However, this effect was mainly associated with compensatory responses based on cell elongation in cucumber, tomato, and onion roots, without a proportional increase in cell proliferation (Figures 2 and 3). This growth pattern resulted in longer and mechanically more fragile roots, as observed at all tested OC + MeP concentrations (Figures 2(m), 2(o), and 3(j)). These structural characteristics indicate cellular stress and may reduce the functional efficiency of the root system during plant development [45]. For the OC + BuP mixture, BuP stands out as the primary agent responsible for the observed root toxicity, since its cytotoxic and genotoxic effects (Figures 3(h) and 3(i)) persisted even in the presence of the sunscreen. This demonstrates that the presence of OC did not mitigate BuP’s toxic action, suggesting a risk of this mixture to plants.

Although the experiments were conducted in aqueous systems, the tested concentrations are environmentally relevant considering the partitioning behavior of micropollutants in soils. OC, MeP, and BuP have been detected in environmental matrices at concentrations ranging from ng·L^−1^ to µg ·L^−1^ and exhibit contrasting hydrophobicity, with log K_OW_ values ranging from ∼2 to ∼7 [18, 50, 51]. This gradient leads to differences in sorption behavior, with more hydrophobic compounds showing greater affinity for soil organic matter, while less hydrophobic compounds exhibit more moderate sorption, consistent with partitioning principles in soil–water systems [84, 85]. Nonetheless, a fraction of these compounds remains in the soil solution—the aqueous phase surrounding soil particles and roots—typically at concentrations on the order of ng·L^−1^, where they may be bioavailable to plants, particularly during germination and early root development [86, 87].

This study provides valuable information on the effects of OC + MeP and OC + BuP on root systems, demonstrating significant alterations in root structure and function in *C. sativus*, *L. esculentum*, and *A. cepa*. Although the experiments were conducted under controlled aqueous conditions, which do not fully replicate the complexity of agricultural soils, the results offer meaningful insight into the potential impacts of the bioavailable fraction of these micropollutants on early plant development. Future studies under soil and field conditions, involving different species and developmental stages, are needed to further expand understanding of the effects of these compounds in mixture on cultivated plants.

## 5. Conclusion

The OC + MeP and OC + BuP mixtures induced oxidative stress in root meristems of *A. cepa* via distinct pathways, resulting in cytotoxic and genotoxic effects with significant aneugenic and clastogenic activities.

The OC + BuP mixture promoted a significant reduction in root growth in *C. sativus*, *L. esculentum*, and *A. cepa*. At the same time, OC + MeP stimulated root elongation in these plants, which was associated with the formation of mechanically fragile roots.

There was a synergistic interaction between OC and MeP and an additive interaction between OC and BuP, with BuP as the primary agent responsible for the mixture’s toxicity.

The results indicate that the combined exposure to OC and parabens, even at environmentally relevant concentrations, may compromise root system functionality and the early developmental performance of cultivated plants. Such alterations may potentially affect crop establishment in agricultural soils contaminated with these micropollutants.

## Funding

This work was supported by the National Council for Scientific and Technological Development (CNPq), 401193/2025‐0.

## Conflicts of Interest

The authors declare no conflicts of interest.

## Data Availability

Data sharing is not applicable to this article as no datasets were generated or analyzed during the current study.
